# GA_3_ is superior to GA_4_ in promoting bud endodormancy release in tree peony (*Paeonia suffruticosa*) and their potential working mechanism

**DOI:** 10.1186/s12870-021-03106-2

**Published:** 2021-07-05

**Authors:** Zhang Yuxi, Yuan Yanchao, Liu Zejun, Zhang Tao, Li Feng, Liu Chunying, Gai Shupeng

**Affiliations:** 1 College of Life Sciences, Qingdao Agricultural University, Qingdao, 266109 China; 2 University Key Laboratory of Plant Biotechnology in Shandong Province, Qingdao, 266109 China; 3 College of Landscape Architecture and Forestry, Qingdao Agriculture University, Qingdao, 266109 Shandong China

**Keywords:** Tree peony, Dormancy release, GAs treatment, GA signal transduction, Starch and sucrose metabolism, DNA replication

## Abstract

**Background:**

Sufficient low temperature accumulation is the key strategy to break bud dormancy and promote subsequent flowering in tree peony anti-season culturing production. Exogenous gibberellins (GAs) could partially replace chilling to accelerate dormancy release, and different kinds of GAs showed inconsistent effects in various plants. To understand the effects of exogenous GA_3_ and GA_4_ on dormancy release and subsequent growth, the morphological changes were observed after exogenous GAs applications, the differentially expressed genes (DEGs) were identified, and the contents of endogenous phytohormones, starch and sugar were measured, respectively.

**Results:**

Morphological observation and photosynthesis measurements indicated that both GA_3_ and GA_4_ applications accelerated bud dormancy release, but GA_3_ feeding induced faster bud burst, higher shoot and more flowers per plant. Full-length transcriptome of dormant bud was used as the reference genome. Totally 124 110 459, 124 015 148 and 126 239 836 reads by illumina transcriptome sequencing were obtained in mock, GA_3_ and GA_4_ groups, respectively. Compared with the mock, there were 879 DEGs and 2 595 DEGs in GA_3_ and GA_4_ group, 1 179 DEGs in GA_3_ vs GA_4_, and 849 DEGs were common in these comparison groups. The significant enrichment KEGG pathways of 849 DEGs highlighted plant hormone signal transduction, starch and sucrose metabolism, cell cycle, DNA replication, etc. Interestingly, the contents of endogenous GA_1_, GA_3_, GA_4_, GA_7_ and IAA significantly increased, ABA decreased after GA_3_ and GA_4_ treatments by LC–MS/MS. Additionally, the soluble glucose, fructose and trehalose increased after exogenous GAs applications. Compared to GA_4_ treatment, GA_3_ induced higher GA_1_, GA_3_ and IAA level, more starch degradation to generate more monosaccharide for use, and promoted cell cycle and photosynthesis. Higher expression levels of dormancy-related genes, *TFL*, *FT*, *EBB1*, *EBB3* and *CYCD,* and lower of *SVP* by GA_3_ treatment implied more efficiency of GA_3_.

**Conclusions:**

Exogenous GA_3_ and GA_4_ significantly accelerated bud dormancy release and subsequent growth by increasing the contents of endogenous bioactive GAs, IAA, and soluble glucose such as fructose and trehalose, and accelerated cell cycle process, accompanied by decreasing ABA contents. GA_3_ was superior to GA_4_ in tree peony forcing culture, which might because tree peony was more sensitive to GA_3_ than GA_4_, and GA_3_ had a more effective ability to induce cell division and starch hydrolysis. These results provided the value data for understanding the mechanism of dormancy release in tree peony.

**Supplementary Information:**

The online version contains supplementary material available at 10.1186/s12870-021-03106-2.

## Background

Bud dormancy of woody perennial plants is an adaptive mechanism that allows them to survive in winter. The annual dormancy is classified into paradormancy, endo dormancy, and ecodormancy based on the internal or external repression signals [1]. In general, environmental signals, such as low temperatures and short-day control endodormancy induction and maintenance [2]. The effective accumulation of low temperature and several chemicals, such as hydrogen cyanamide (HC), mineral oil, potassium nitrate, gibberellin (GA) and 5-azacytidine (5-azaC), could promote dormancy release and induce bud break of deciduous trees in temperate areas [3–5].

Tree peony (*Paeonia suffruticosa* Andrews), originated from China, is well known as a horticultural plant in the world for its beautiful flower type and color. Tree peony has a short and concentrated florescence every year, so anti-season culture forms a new industrial chain and occupies a great part in tree peony industry, and the key problem is to break bud dormancy, which is necessary to ensure the quality of flowering. As well as other perennial plants, like sweet cherry, grape, apple, peach, and kiwifruit [6–10], the bud dormancy of tree peony is a biological characteristic and also a necessary process before bud sprouting and flowering [11].

The buds require sufficient chilling to break endodormancy and transit into the ecodormant state, then they will burst when the comfortable environment returns. GAs had been demonstrated to play important roles in the activity-dormancy-activity cycle [4, 12–14]. For example, Nell et al. found that foliar applications of GA_3_ or GA_4,7_ accelerated the flowering and increased the pedicel length and flower size in azalea [15]. In flower buds of Japanese apricot, GA_4_ significantly accelerated dormancy release, and the promotion of dormancy release was related to energy metabolism activation by proteomic and transcriptomic analysis [16]. When *Paeonia lactiflora* endured insufficient chilling accumulation, the GA_3_ treatment effectively broke dormancy, accelerated sprouting and promoted subsequent growth and flowering [17]. In summary, different kinds of gibberellins show the inconsistent effect on breaking dormancy in various plants. Then, which gibberellin (GA_3_ or GA_4_) usually used in production is more effective to break bud dormancy of tree peony?

Recent reports showed that several genes associated with GA synthesis and signal transduction were involved in bud dormancy regulation. Combination of transcript profiling and microarray analysis, Gai et al. found that the activation of GA pathway played a core role in the regulation of tree peony bud dormancy release [18]. It is well known that the key genes associated with gibberellin synthesis and signaling are GA 20-oxidase gene (*GA20ox*), GA 3-oxidase gene (*GA3ox*), *GA20ox, GA3ox, GA2ox, GID1, DELLA* and *GID2* [19]. Bioactive GAs is synthesized through complex pathways, of which GA20ox and GA3ox are the key rate-limiting enzymes [20]. In tea, the differentially-expressed *genes (DEGs), CsGA3ox and CsGA20ox*, played an important role in regulating the bud activity-dormancy transition [14]. Conversely, *PmRGL2* encoding a DELLA protein acted as a negative regulator of dormancy release through GA signaling pathway in Japanese apricot [21]. The F-box protein GID2, a subunit of the SCF E3 ubiquitin ligase complex, recognizes the GA-GID1-DELLA complex and helps to degrade the DELLA through the 26S proteasome, leads to de-repression and transcriptive activation of GA-responsive genes [20].

Abscisic acid (ABA) is generally believed to be another core hormone to maintain and regulate bud dormancy with antagonistic effect to GA [12]. The increasement of the ABA content benefits to induce dormancy in autumn, while its content decrease is the trigger for dormancy release in poplar, grape and pear [10, 22–25]. In poplar, ABA content increased after short day exposure and reached a peak after growth cessation in the apex, and the key genes involving in ABA biosynthesis and ABA signal transduction were also induced [26]. ABA bounds to members of the PYL/RCAR ABA receptor family that initiates signal transduction inhibiting type 2C protein phosphatases [27]. The ABA-responsive element (ABRE) is the major cis-element for ABA-responsive gene expression, and the ABRE-dependent gene expression is regulated by ABRE-binding factor (ABF). In peach, PpABF2 interacted with PpTCP20 to form heterodimers and regulate bud endodormancy. PpyABF3 could bind to the promoter of *PpyDAM3* to activate its expression, while PpyABF2 interrupted this activation role by binding with PpyABF3 during the pear endodormancy maintenance [9]. Recent report showed that *SHORT VEGETATIVE PHASE* (*SVP*)*-*like (*SVL*) played a vital role in the dormancy of poplar. Prolonged chilling deceased ABA contents and *SVL* expression, resulting in the induction of *FT1* expression and GA biosynthesis, which accelerated dormancy release [28].

Bud dormancy-growth transition is accompanied by many metabolic and developmental processes, including carbohydrate metabolism, photosynthesis, cell division, etc. As early as 1990, Bonicel et al. indicated that deep dormancy of poplar bud was characterized by low carbohydrate levels, and the end of dormancy was marked by major changes that starch levels declined and sugars rose [29]. During dormancy release of Japanese pear, a decrease of starch content in the shoot and an increase of soluble sugars in both the flower bud and the shoot were observed [30]. Starch, a polymer of glucose molecules, is the predominant form of carbohydrate storage in cells. In poplar, the activation of carbohydrate metabolism-related proteins mainly occurred at the stage of endodormancy release [31]. Carbohydrate metabolism activation and variation of plant hormone signal transduction were also found in the dormancy release process of *Lilium pumilum* [32] and tree peony [33]. In addition, the cell proliferation-related genes including MAP kinase, cyclins, and cyclin dependent-kinases, etc., were assumed to involve in the dormancy-growth transition. In grapevine, cell cycle genes encoding cyclin dependent-kinase (CDKB1 and CDKB2) and cyclins (CYCA, CYCB and CYCD) were activated during bud endodormancy release [34]. These results indicated that dormancy break was accompanied by cell cycle and cell division reactivation.

Additionally, recent studies have identified several dormancy release related genes. FLOWERING LOCUS T (FT) was believed to play vital role in dormancy release [35], while its paralog CENTRORADIALIS/ TERMINAL FLOWER 1 (CEN/TFL1) acted as a negative regulator of dormancy release in poplar. *TFL* was also assumed to be a marker of bud burst [35, 36]. AP2 type transcript factor EBB1 (EARLY BUD-BREAK 1) accelerated dormancy release and bud break, and EBB3 was part of the EBB1/SVL-mediated regulatory mechanism, which promoted the expression of *CYCD3.1* and positively regulated bud-break with activation of cell cycle in poplar. *SVL* involved in the same pathway with a negative manner [22, 37].

In this study, the morphological changes indicated that both GA_3_ and GA_4_ greatly accelerated bud dormancy release, but the effect of GA_3_ was better than that of GA_4_ to accelerate bud burst and subsequent growth in tree peony. In order to understand how exogenous GA_3_ or GA_4_ affected dormancy release, PacBio full-length sequencing in the dormant bud of tree peony was performed to obtain high-quality transcriptome data as reference genome, the DEGs of tree peony buds with exogenous GA_3_ or GA_4_ application were obtained by illumina transcriptome sequencing, and the contents of sugar and endogenous phytohormones were measured, respectively. All results would provide valuable data for the mechanism of dormancy release in tree peony.

## Results

### Morphology changes after exogenous GA_3_ and GA_4_ treatments

**Fig. 1:**
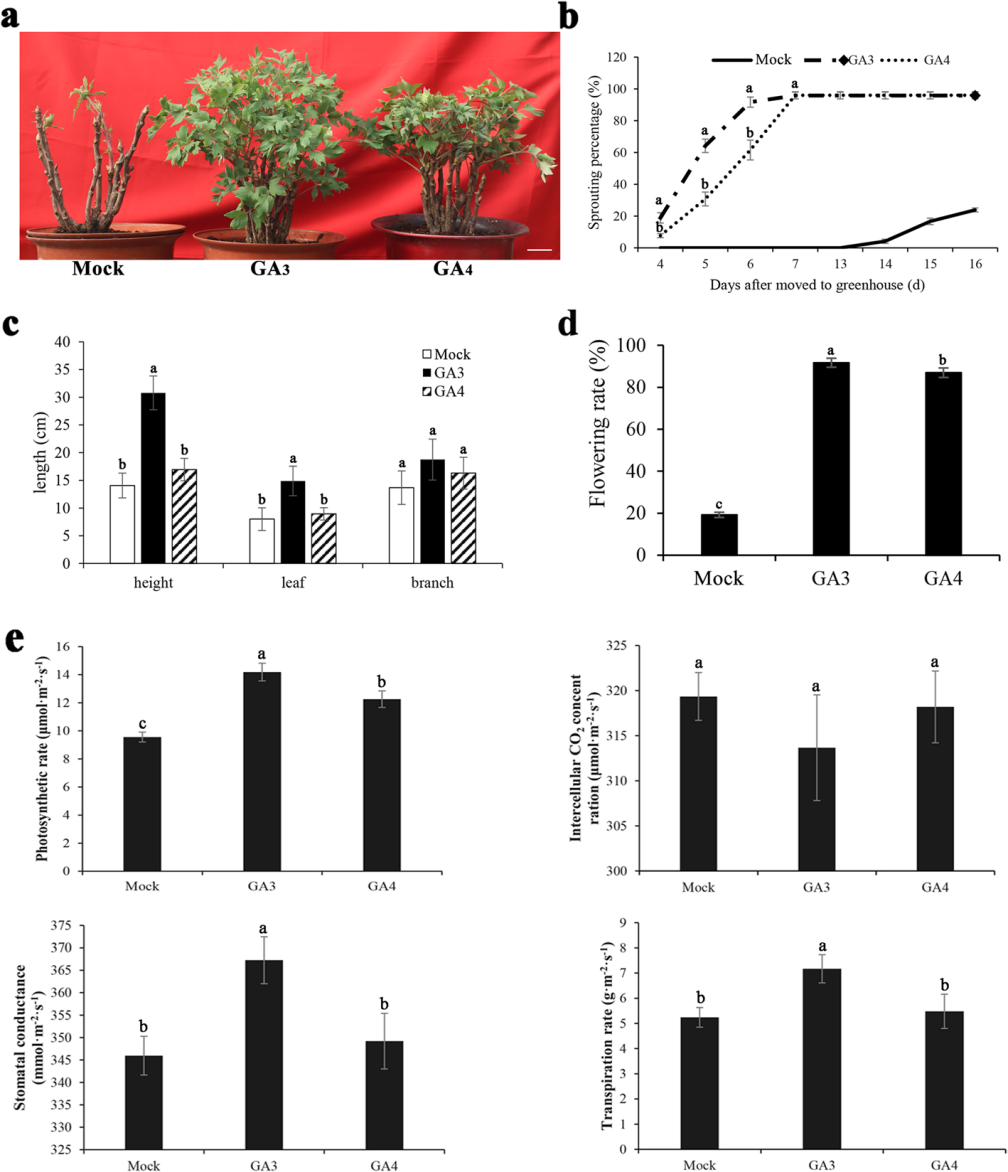
Effect of GA_3_ and GA_4_ applications in *P. suffruticosa* cv ‘Luhehong’. **a** Morphological observation after being transferred into greenhouse for 16 d by exogenous GA_3_ and GA_4_ applications, the samples treated by sterile distilled water was used as mock. Bar = 4 cm; **b** The sprouting percentage after being transferred into greenhouse for 16 d by exogenous GA_3_ and GA_4_ applications; **c** Branch height of shoots, the length of the first leaf and flowering rate after being transferred into greenhouse for 16 d by exogenous GA_3_ and GA_4_ applications; **d** Flowering rate after being transferred into greenhouse for 40 d by exogenous GA_3_ and GA_4_ applications. **e** Effect of GAs treatment on the photosynthesis of tree peony ‘Luhehong’ leaves. Duncan’s test at *p* < 0.05 after analysis of variance; data were shown as mean ± SD in three biological replicates. The lowercase letters on the histogram represented the differentiation

Despite past study reported that GA application could effectively promote endodormancy release and accelerate bud sprouting in tree peony [38], the effects of different GAs were still unknown. Therefore, GA_3_ and GA_4_, commonly used in production, were applied to compare their effects on dormancy release and subsequent growth. Exogenous GA_3_ and GA_4_ induced the bud burst after 4 d applied, and almost all apical buds sprouted 3 d thereafter, but it was 1 d earlier in GA_3_ group than in GA_4_ group to reach the peak. In contrast, the sprouting rate in mock group was 19.24% after 16 d, 96.20% in GA_3_ treated group (*p* = 0.000), and 95.82% in GA_4_ treated group (*p* = 0.000) (Fig. 1b). After GA_3_ and GA_4_ treated for 16 d, their morphology was investigated, including the shoot height, the length of branch and leaf, and flowering rate. GA_3_ and GA_4_ feedings significantly promoted the leaf length, shoot height and flowering rate (Fig. 1a, c). Compared with the mock group, GA_3_ application increased the height of shoot (average 30.83 cm, *p* = 0.014) and the length of leaf (average 14.89 cm, *p* = 0.010), and its effect was better than GA_4_, while the difference of branch length was not significant in GA_3_ (*p* = 0.376) and GA_4_ (*p* = 0.373) treated groups (Fig. 1c). Additionally, the flowering rate induced by GA_3_ was significantly higher than that by GA_4_ treatment (*p* = 0.039) (Fig. 1d). Altogether, these changes of morphology indicated that both GA_3_ and GA_4_ applications effectively accelerate bud dormancy release, burst, growth and flowering, but GA_3_ was superior to GA_4_.

As known, photosynthesis is an energy resource for shoot growth and flowering. Therefore, the photosynthetic parameters in the leaves after GA_3_ and GA_4_ feedings for 16 d were measured, respectively. Compared to mock group, photosynthetic rate significantly increased after GA_3_ (*p* = 0.000) and GA_4_ (*p* = 0.002) treatments. The stomatal conductance and transpiration rate by GA_3_ application (*p* = 0.006) were greater than that in mock group, whereas there was no difference between GA_4_ treatment and mock (*p* = 0.504). In comparison with mock group, the intercellular CO_2_ concentrations were not different among GA_3_ (*p* = 0.204) and GA_4_ (*p* = 0.693) groups (Fig. 1e).

### The PacBio full-length transcriptome in tree peony during chilling-induced dormancy release

**Fig. 2:**
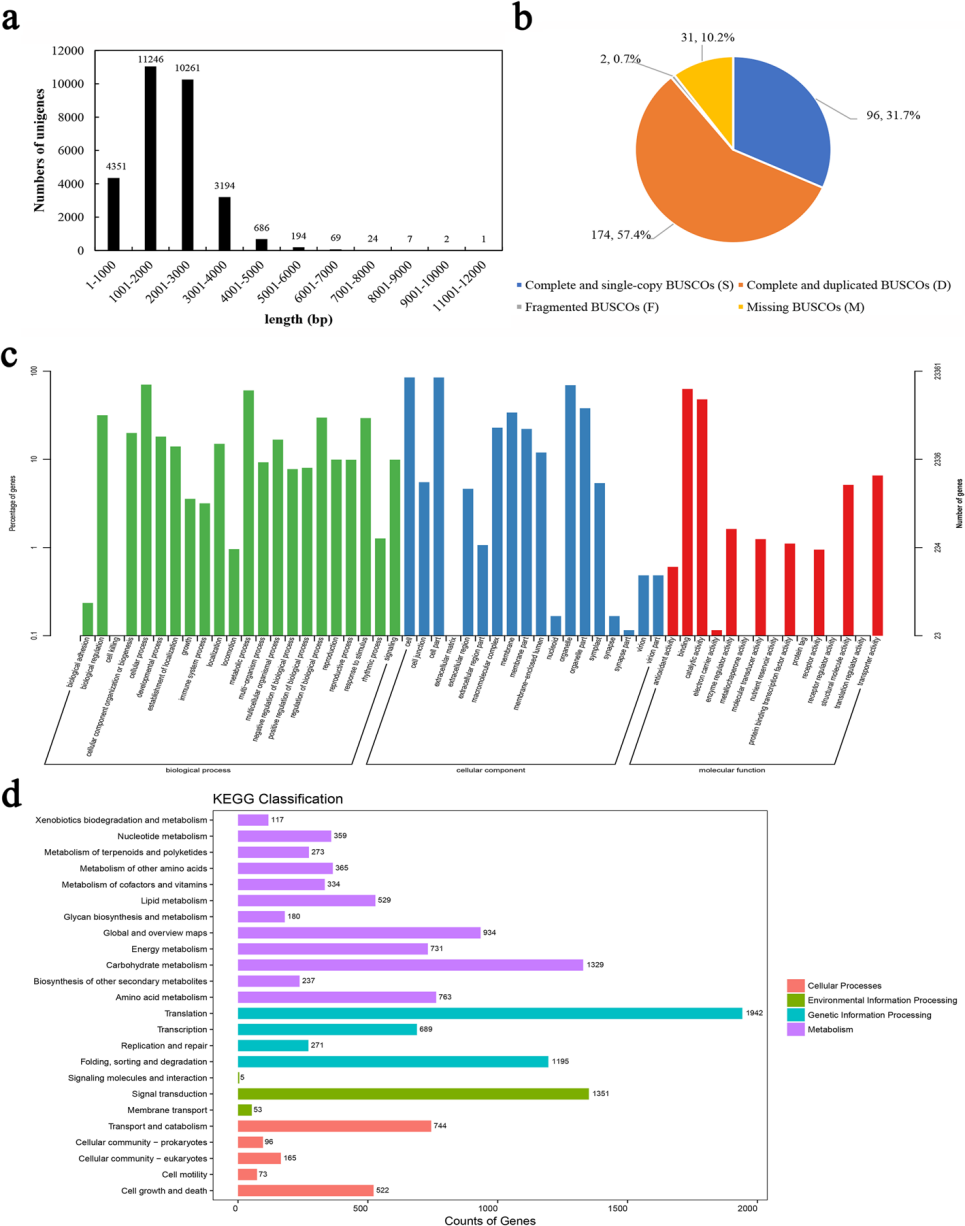
The length distribution, BUSCO analysis of transcript completeness, GO analysis and KEGG pathway classification of the assembled unigenes in *P. suffruticosa* cv ‘Luhehong’. **a** The length distribution of the assembled unigenes; **b** BUSCO analysis of transcript completeness; **c** GO analysis of the assembled unigenes; **d** KEGG pathway classification of the assembled unigenes

According to previous data, the samples after 0, 7, 14, 21 and 28 d chilling durations represented the whole process from endodormancy, endodormancy release to ecodormancy. Until now, there is still no high-quality genome data of tree peony. Therefore, the samples of different chilling durations (0, 7, 14, 21 and 28 d) were mixed and used to construct a pool to obtain a wide coverage of the tree peony transcriptome. A total of 18 470 579 reads were obtained with the max length of 200 013 bp and the min length of 50 bp, and the mean length was 1 778 bp. After removing the low-quality raw reads through the three steps including CCS, the classify and cluster (Isoseq) (https://github.com/PacificBiosciences/IsoSeq_SA3nUP wiki# datapub), 38 288 high-quality sequences with the length ranged from 79 bp to 11 471 bp were finally obtained. Of them, the quantity of reads with the length between 1 001 and 2 000 bp (11 246) was the most, followed by the reads with the length between 2 001 and 3 000 bp (10 261) (Fig. 2a). The generated isoforms were then filtered with CD-HIT software based on an identify of 98%. The full-length transcript completion was examined using eukaryote_odb9 database containing single-copy gene sets of 100 species. After removing redundancy, there were 89.1% full-length transcripts matched to the complete BUSCO database, of which the number of complete single-copy unigenes accounted for 31.7%, complete duplicated unigenes for 57.4%, the fragmented unigenes for 0.7%, and the quantity of the missing unigenes for 10.2% (Fig. 2b). Finally, total 30 035 non-redundant full-length unigenes were obtained and used for the subsequent analysis.

The 30 035 non-redundant unigenes were performed to annotate in different public databases, and most of them were longer than 1 000 bp (Additional file 1). A total of 23 361 unigenes were annotated to GO classification terms, and there were 19 781, 21 120 and 19 843 unigenes classified into biological process, cellular component, and molecular function category, respectively (Fig. 2c). In the “biological process” category, the unigene numbers classified into cellular process (16 435), metabolic process (14 128) and biological regulation (7 388) were in the majority. In the “cellular component” category, the majority of the unigenes were represented by cell (19 827), cell part (19 802) and organelle (16 200). Terms of binding (14 670) and catalylic activity (11 185) occupied a clear majority in “molecular function” category. In total, 13 155 unigenes annotated in KEGG database, and the most majority of unigene were enriched in translation (1 942), signal transduction (1 351), carbohydrate metabolism (1 329) and folding, sorting and degradation (1 195) (Fig. 2d). In addition, there were 28 420 (94.62%), 27 609 (91.92%), 25 071 (83.47%), and 18 379 (61.19%) unigenes matched sequences in the Nr, eggnog, Swissprot, and KOG databases, respectively.

In addition, the annotated unigenes were blasted to the transcription factor (TF) database, and total 8 019 unigenes were found to encode potential TFs (Additional file 2). Most of the TFs were mainly distributed in the families of C3H, MADS, FAR1, PHD, bHLH, MYB-related, FHA, C2H2, NAC, AP2-EREBP, etc. The opening reading frames (ORFs) of these assembled unigenes were predicted by TransDecoder with priority order of Nr, Swissprot and KOG, and hit total 29 418 unigenes, occupied by 97.9% of the non-redundant unigenes. The results indicated that the transcriptome data was high quality and informative.

### Illumina transcriptome sequencing in tree peony after exogenous GA_3_and GA_4_ treatments

As indicated above, exogenous GA_3_ and GA_4_ had a different effect on dormancy release and subsequent growth, and we wanted to know what molecular mechanism hid in this process? In order to answer this question, the illumina transcriptome sequencing of mock, GA_3_, and GA_4_ treated buds were carried out. After quality control, a total of 144.55 M, 143.21 M and 144.03 M clean reads were screened out from 148.19 M, 147.94 M, and 147.66 M raw reads, respectively. The average Q30 percentage (percentage of bases whose quality was greater than 30 in clean reads) and GC percentage were 94.32% and 45.14%, respectively (Table 1). Totally 124 110 459, 124 015 148 and 126 239 836 reads in mock, GA_3_ and GA_4_ groups were mapped to the above reference unigenes, and the average mapped percentages were 85.86%, 86.59% and 87.65%, respectively (Table 1).

**Table 1:** Summary of the Illumina sequencing output for flower buds after exogenous GA_3_ and GA_4_ applications

Sample	Raw Reads (M)	Raw Bases (G)	Clean Reads (M)	Clean Bases (G)	Valid bases (%)	Q30 (%)	GC (%)	Total mapped clean reads (the mapped percentage %)
mock_1	49.89	7.48	48.55	6.99	93.45	94.59	45.18	41 541 249 (85.56%)
mock_2	49.04	7.36	47.87	6.96	94.65	94.74	45.21	41 168 265 (86.00%)
mock_3	49.26	7.39	48.13	7.00	94.75	94.87	45.02	41 400 945 (86.01%)
GA_3__1	49.39	7.41	47.77	6.85	92.51	93.29	45.08	41 188 415 (86.22%)
GA_3__2	48.99	7.35	47.36	6.79	92.45	93.13	45.12	41 025 753 (86.62%)
GA_3__3	49.56	7.43	48.08	6.90	92.75	93.46	45.12	41 800 980 (86.94%)
GA_4__1	48.99	7.35	47.63	6.88	93.69	94.76	45.07	41 486 416 (87.11%)
GA_4__2	49.45	7.42	48.38	7.00	94.37	95.07	45.18	42 446 359 (87.73%)
GA_4__3	49.22	7.38	48.02	6.95	94.14	94.94	45.30	42 307 061 (88.10%)

Notes: Mock, sterile distilled water treatment for 48 h; GA_3_, GA_3_ treatment for 48 h; GA_4_, GA_4_ treatment for 48 h

### DEGs after exogenous GA_3 _and GA_4_ treatments

In order to investigate the repeatability among biological replicates and the correlation between different samples, PCA analysis was performed based on the transcription abundances of all the unigenes (Additional file 3). The result indicated that biological triplicates had high reliability for each of the repeats grouped together, respectively.

A large number of DEGs were obtained using DEGseq software in the comparisons of GA_3_ vs mock, GA_4_ vs mock, GA_3_ vs GA_4_ (Table 2). Compared with the mock group, the DEGs number of GA_4_ treated group was more than that of GA_3_ treated group. In GA_3_ vs mock, totally 879 DEGs were obtained, including 405 up-regulated and 474 down-regulated DEGs, respectively. In GA_4_ vs mock, there were 2 595 DEGs, including 1 187 up-regulated and 1 408 down-regulated DEGs, respectively. Between GA_3_ and GA_4_ treatment, a total of 1 179 DEGs were obtained, of which 547 up-regulated and 632 down-regulated DEGs.

**Table 2:** Statistics of the DEGs in different comparisons

Comparisons	Up-regulated DEGs (%)	Down-regulated DEGs (%)	Total DEGs (*p*-value < 0.05&|log2FC|> 1)
GA_3_ vs mock	405 (46.07%)	474 (53.93%)	879
GA_4_ vs mock	1 187 (45.74%)	1 408 (54.26%)	2 595
GA_3_ vs GA_4_	547 (46.40%)	632 (53.60%)	1 179

**Fig. 3:**
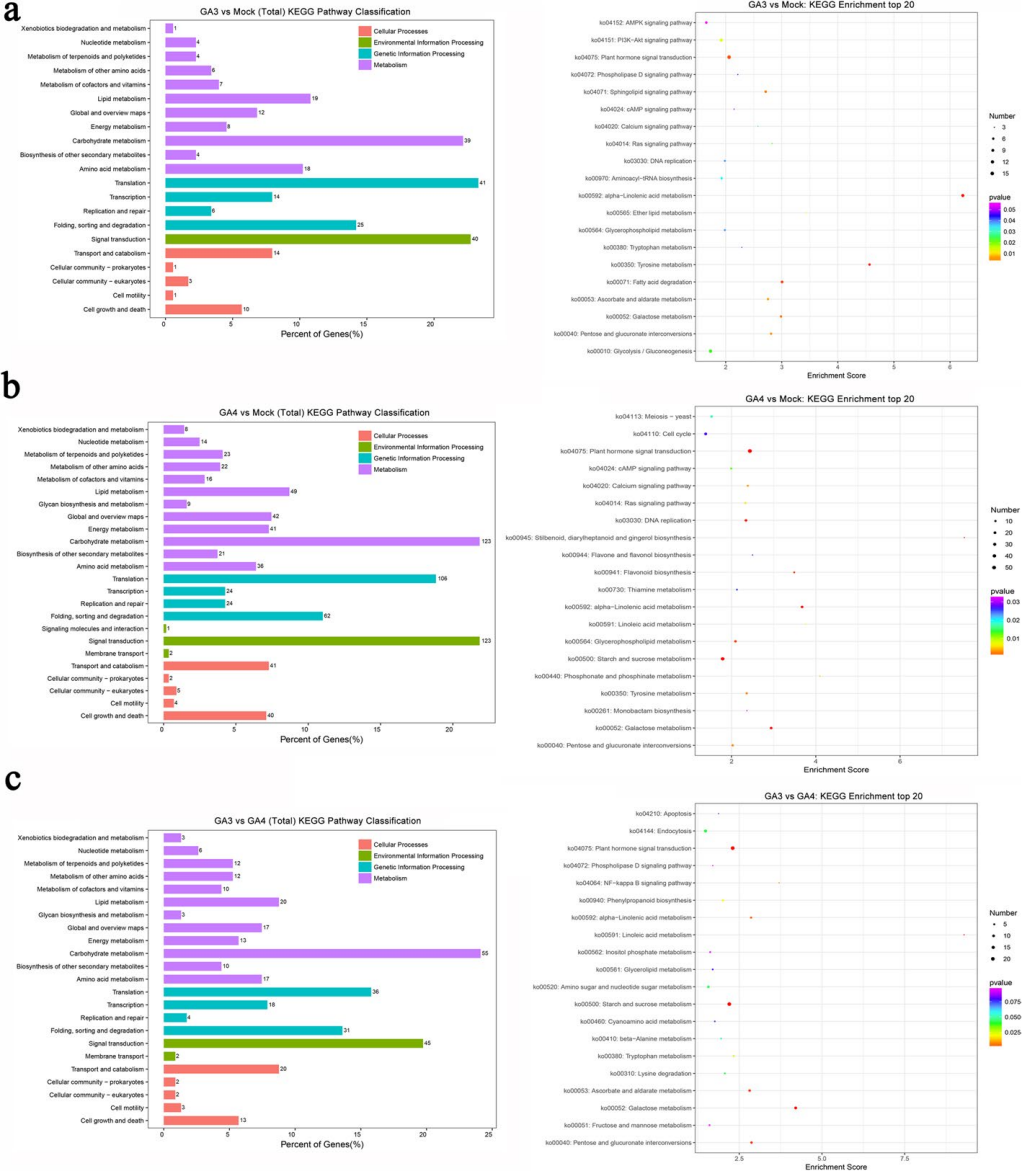
KEGG pathway classification and KEGG enrichment of DEGs (*p *-value < 0.05 & |log_2_FC|> 1) after GA_3_ and GA_4_ applications. **a** GA_3_ vs mock; **b** GA_4_ vs mock; **c** GA_3_ vs GA_4_

The GO classification and KEGG enrichment analysis of DEGs were further performed. Totally 652, 870, and 1 922 DEGs were divided into 49, 50, and 49 sub-categories in GA_3_ vs mock, GA_4_ vs mock, and GA_3_ vs GA_4_, respectively. (Additional file 4). Among these three comparison groups, the major sub-categories were “cellular process” in “biological process” category with 1 260, 474 and 592 DEGs, “cell” in “cellular component” category with 1 445, 433, and 626 DEGs, and “binding” in “molecular function” category with 1 167, 427, and 526 DEGs, respectively. A total of 227, 838, and 354 DEGs were annotated by the KEGG database and classified into 21, 24 and 23 sub-categories, respectively (Fig. 3). In the top 20 KEGG enrichment terms, many of the DEGs were enriched in plant hormone signal transduction with 15, 57, and 22, starch and sugar metabolism with 11, 44 and 22, respectively (Fig. 3).

**Fig. 4:**
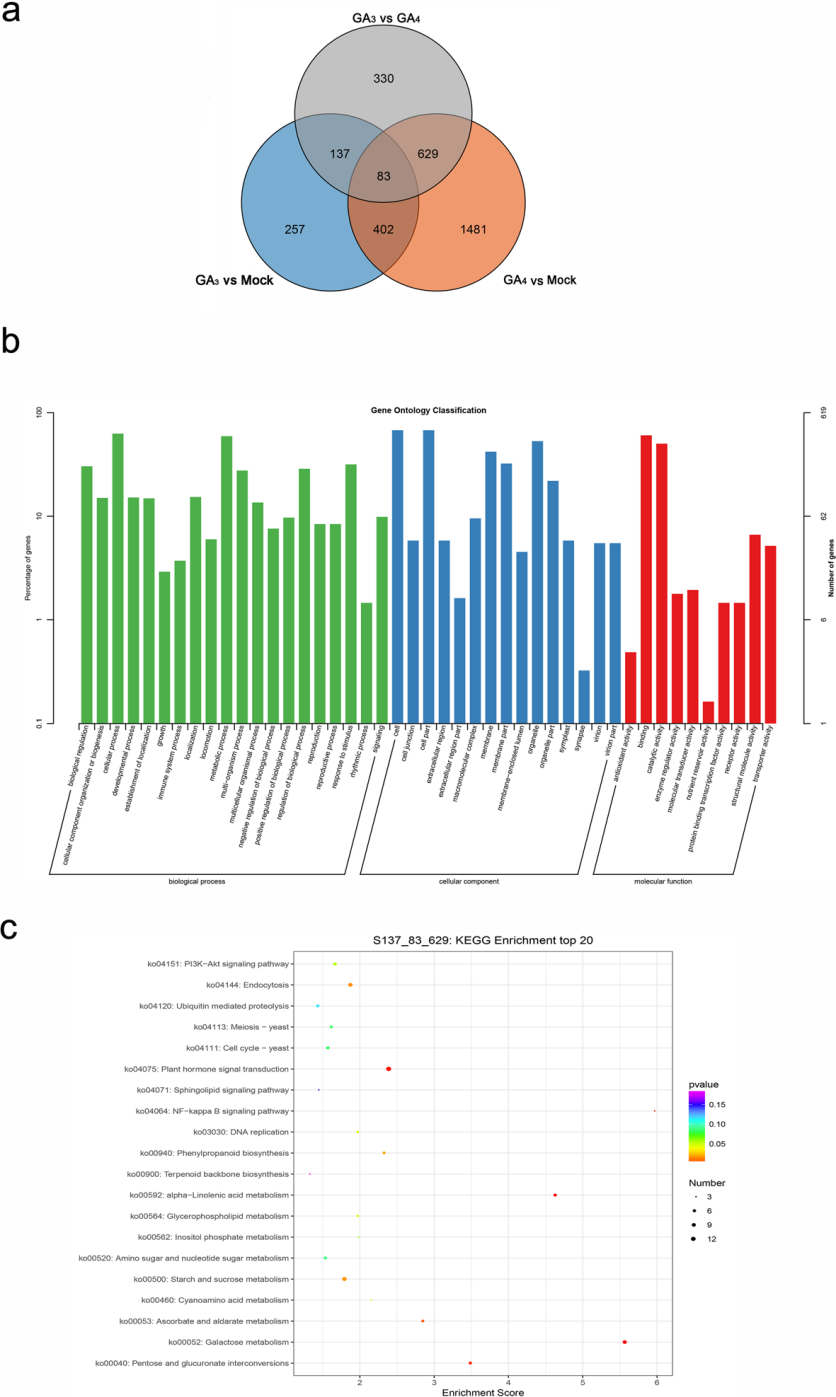
GO classification and KEGG enrichment of the 849 overlapping DEGs between GA_3_ vs GA_4_ and GA_3_ vs mock, GA_3_ vs GA_4_ and GA_4_ vs mock, respectively. **a** Venn diagram of DEGs among different comparison groups; **b** GO classification of the 849 (137 + 83 + 629) overlapping DEGs between GA_3_ vs GA_4_ and GA_3_ vs mock, GA_3_ vs GA_4_ and GA_4_ vs mock; **c** KEGG enrichment of the 849 (137 + 83 + 629) overlapping DEGs between GA_3_ vs GA_4_ and GA_3_ vs mock, GA_3_ vs GA_4_ and GA_4_ vs mock

In order to know the reason for exogenous GA_3_ and GA_4_ effectively accelerating bud dormancy release and burst, and their different effects on plant morphological changes, a Venn diagram was constructed to show the overlapping DEGs (Fig. 4a). There were 220 and 712 DEGs overlapping between GA_3_ vs GA_4_ and GA_3_ vs mock, GA_3_ vs GA_4_ and GA_4_ vs mock, respectively, and 83 DEGs were common in these two comparison groups. Among the overlapping 849 DEGs, a total of 619 were assigned to GO terms, and the major sub-categories were “cellular process” and “metabolic process” in “biological process” category with 389 and 367 DEGs, “cell” and “cell part” in “cellular component” category with 419 and 419 DEGs, and “binding” and “catalytic activity” in “molecular function” category with 374 and 311 DEGs, respectively (Fig. 4b). KEGG pathway enrichment of these 849 overlapping DEGs was performed, and they were significantly enriched in 11 KEGG pathways (*p* < 0.05), including “plant hormone signal transduction” with 14 DEGs, “starch and sucrose metabolism” with 11 DEGs, “endocytosis” with 10 DEGs, “Galactose metabolism” with 9 DEGs, “alpha-Linolenic acid metabolism” with 6 DEGs, “pentose and glucuronate intercom” with 6 DEGs, “Cell cycle” with 6 DEGs, “Ascorbate and aldarate metabolism” with 5 DEGs, “Phenylpropanoid biosynthesis” with 5 DEGs, “DNA replication” with 4 DEGs etc. (Fig. 4c).

### Identification of plant hormone and signaling transduction, sugar metabolism, and cell cycle-related DEGs after GA_3 _and GA_4_treatments

**Fig. 5:**
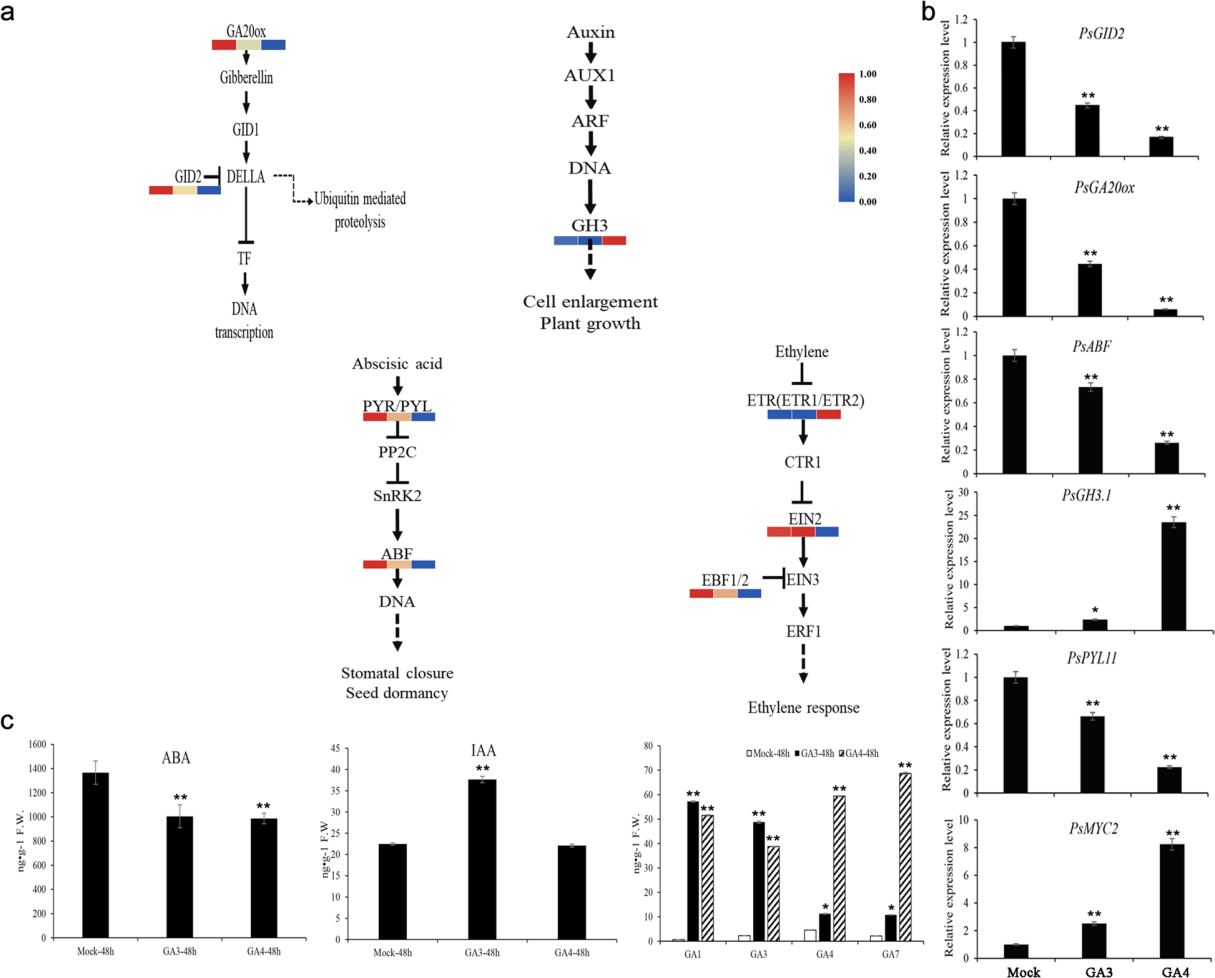
The relative expression levels of plant hormone signal transduction-related DEGs and the contents of endogenous GAs, ABA, and IAA after GA_3_ and GA_4_ applications in *P. suffruticosa* cv ‘Luhehong’. **a** Heatmap of plant hormone signal transduction-related DEGs after GA_3_ and GA_4_ applications based on the RNAseq data. The colors from blue to red in heatmap represented the relative expression level of DEGs from low to high, and the blue-red colors corresponded to the 0–1 normalization (minimum–maximum normalization) values of log_2_(FPKM). **b** The relative expression levels of plant hormone signal transduction-related DEGs after GA_3_ and GA_4_ applications by qPCR. **c** The contents of endogenous GAs, ABA, and IAA after 48 h GA_3_ and GA_4_ feedings, and noted GA_3_-48 h, GA_4_-48 h. The mean ± SD in three biological replicates was shown. *, ** indicated significant differences of one-way ANOVA at *p* < 0.05, *p* < 0.01, respectively

**Fig. 6:**
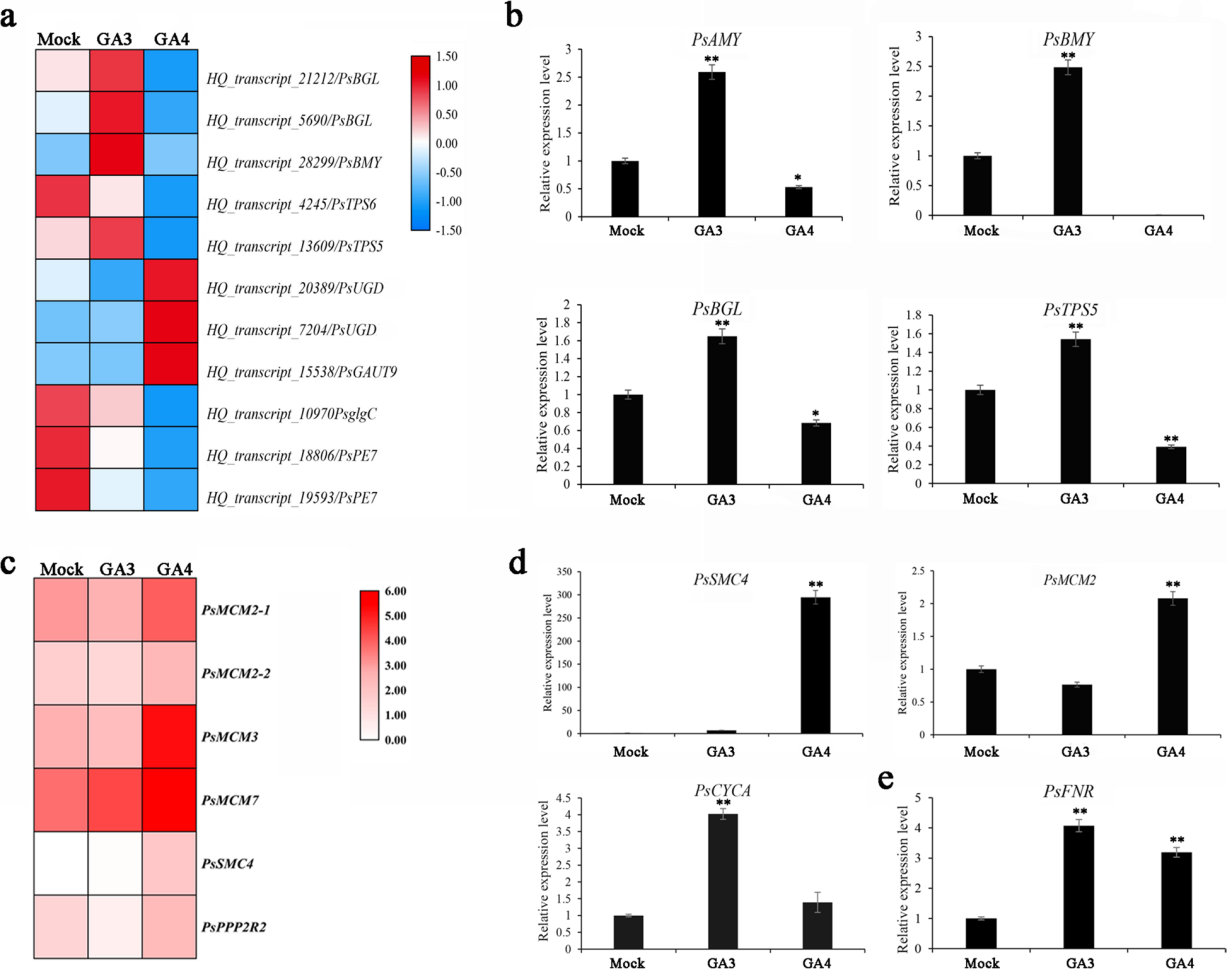
The expression levels of starch and sucrose metabolism and cell cycle-related DEGs after GA_3_ and GA_4_ applications in *P. suffruticosa* cv ‘Luhehong’. **a** Heatmap of starch and sucrose metabolism-related DEGs after GA_3_ and GA_4_ applications based on the RNAseq data. **b** The expression levels of starch and sucrose metabolism -related DEGs after GAs applications by qPCR. **c** Heatmap of cell cycle-related DEGs enriched in KEGG pathway map based on the RNAseq data. **d** The expression levels of cell cycle-related DEGs after GAs applications by qPCR. **e** The expression level of *PsFNR* after GAs applications by qPCR. Data were represented as mean of three different determinations ± SD. Asterisks indicate statistically significant differences (one-way ANOVA, *p < 0.05, **p < 0.01)

Based on the Venn diagram and KEGG enrichment of the overlapping 849 DEGs (Additional file 5) between GA_3_ vs GA_4_ and GA_3_ vs mock, GA_3_ vs GA_4_ and GA_4_ vs mock, we constructed the phylogenetic trees of the related DEGs (Additional file 6), which enriched in the KEGG pathways about plant hormone signal transduction, starch and sucrose metabolism, cell division and DNA replication, and analyzed their expression patterns by the results of RNAseq data and real-time quantitative RT-PCR (qPCR) (Fig. 5, Fig. 6). Totally, 14 DEGs were related to plant hormone and signaling transduction, including GA, ABA, Auxin and ethylene pathway (KEGG: ko04075). The relative expression of the related unigenes validated by qPCR was similar to the RNAseq data (Fig. 5a and b, Additional file 7). Among these DEGs, a F-box protein gene *PsGID2*, encoding a subunit of ubiquitin E3 ligases, decreased after GA_3_ and GA_4_ treatments, and the down-regulated level of GA_4_ treatment was greater. The expression levels of two ABA responsive element binding factor gene (*PsABF*) and three ABA receptor PYR/PYL family members (*PsPYL8, PsPYL12* and *PsPYL1*) were down-regulated after exogenous GAs treated. Among four auxin-related DEGs, two DEGs encoding indole-3-acetic acid-amido synthetase GH3 family members including *PsGH3.6* and *PsGH3.1* significantly increased after GAs applications, of which the expression levels of *PsGH3.1* were increased 2.37-fold by GA_3_ and 24.22-fold by GA_4_ treatment, respectively. RNAseq showed that the expressions of two ethylene receptor 2 (*PsETR2*) were up-regulated after GA_3_ and GA_4_ treatments, with more than 2.28-fold increasing by GA_4_, and the ethylene-insensitive protein gene (*PsEIN2*) was significantly inhibited by GA_4_ treatment. One EIN3-binding F-box protein gene (*PsEBF1*) was significantly down-regulated after GA_4_ application. In addition, a transcription factor encoding gene, *PsMYC2*, was extremely induced by GAs applications (Fig. 5a, b).

The relative expression of starch and sugar metabolism and cell cycle-related DEGs were also validated by qPCR, and the results indicated that the expression profiling by qPCR was consistent with that of RNAseq data (Fig. 6, Additional file 7). Among the 11 starch and sugar metabolism-related DEGs (KEGG: ko00500), starch hydrolase beta-amylase gene *PsBMY* was only induced by GA_3_ treatment, and hardly detected in GA_4_-treated group. Two beta-glucosidase genes (*PsBGLs*) were down-regulated after GA_4_ feeding, but up-regulated by GA_3_ application. *PsTPS5* (trehalose 6-phosphate synthase gene) showed up-regulation by GA_3_ treatment, but down-regulation by GA_4_ treatment, while *PsTPS6* was inhibited by both GA_3_ and GA_4_ treatments. Two *PsUGD*s (*PsUGD1* and *PsUGD3*) encoding UDP glucose 6-dehydrogenase were obviously induced by GA_4_ application. One glucose-1-phosphate adenylyltransferase gene *PsglgC* and two pectinesterase genes (*PsPEs*) were down-regulated both by GA_3_ and GA_4_, respectively. *PsGAUT9*, encoding alpha-1,4-galacturonosyltransferase involving in pectin biosynthesis was dramatically induced by GA_4_ (Fig. 6a, b).

In addition, among 6 DEGs enriched in cell cycle pathway (KEGG: ko04111), structural maintenance of chromosome 4 gene (*PsSMC4*) significantly increased after GA_3_ and GA_4_ treatments, and its expression level increased 286.13-fold after GA_4_ feeding compared to mock. Two DNA replication licensing factor genes *PsMCM2* showed significantly decreased after GA_3_ treatment but increased by GA_4_ feeding (Fig. 6c, d). The expression level of Ferredoxin-NADP^+^ oxidoreductase gene (*PsFNR*), involving in photosynthesis pathway (KEGG: ko00195), significantly up-regulated after GA_3_ and GA_4_ treatments, and its expression level increased 4.15-fold after GA_3_ treatment compared with mock group (Fig. 6e).

### Quantification of endogenous phytohormone, sugar and starch after GA_3 _and GA_4 _applications

Because plant hormone and signal transduction-related DEGs were enriched in KEGG analysis, the contents of endogenous phytohormone, including GAs, ABA, and IAA, were measured. The result showed that the contents of GA_1_, GA_3_, GA_4_ and GA_7_ increased after GA_3_ and GA_4_ treatments, of which the contents of endogenous GA_1_ and GA_3_ increased about 57.19-fold and 48.75-fold after exogenous GA_3_ treated for 48 h, and GA_4_ and GA_7_ increased about 59.37-fold and 68.66-fold after 48 h exogenous GA_4_ feeding. The results indicated that intake of exogenous GAs enhanced the conversion of bioactive GAs. Additionally, the transcript of *PsGA20ox*, the key enzyme of gibberellin synthesis, was analyzed by qPCR (Fig. 5b). The expression levels of *PsGA20ox* were down-regulated by both GA_3_ and GA_4_ applications, and which decreased 5.92-fold by GA_4_, suggesting the exogenous application of GAs depressed their endogenous synthesis and feedback regulation. The contents of endogenous ABA significantly decreased after GA_3_ and GA_4_ treatments, which were consistent with the down-regulated expression of ABA receptor (*PsPYL1*, *PsPYL8*, *PsPYL12*) (Fig. 5b, c). In addition, the contents of endogenous IAA were up-regulated by GA_3_, but there was no significant difference between GA_3_ and GA_4_ application.

The results of RNAseq and qPCR showed that *PsBMY* was only induced by GA_3_, and the expressions of *PsBGLs* and *PsAMY* significantly increased by GA_3_ (Fig. 6a, b). Starch contents significantly decreased after GA_3_ and GA_4_ feedings evaluated by anthrone colorimetry method, and more dramatically in GA_3_ group, which suggested that GA_3_ application induced more polysaccharides to be hydrolyzed. To verify these, the soluble sugar contents of different components were determined by GC–MS/MS (Table 3). Totally, 13 different soluble sugar components besides lactose were determined. Among all these sugar components, sucrose was the most abundant among disaccharides, and its content decreased after both GA_3_ and GA_4_ applications. The contents of inositol, fructose and glucose were affluent among 9 monosaccharides, and glucose and fructose significantly increased in GA_3_ and GA_4_ treated groups. Interestingly, the content of inositol was significantly increased by GA_3_ but decreased by GA_4_ application. Additionally, the amounts of galactose, rhamnose and fucose significantly increased only after GA_3_ application, while maltose was significantly induced only by GA_4_. The content of sorbose rose, but no significant difference between GA_3_ and GA_4_.

**Table 3:** Contents of different soluble sugar components and starch in tree peony bud after GA_3_ and GA_4_ applications

Component	Type	mock (mg·g^−1^)	GA_3_ (mg·g^−1^)	GA_4_ (mg·g^−1^)
Starch	polysaccharides	100.0100 ± 0.8900a	85.8900 ± 1.9200c	94.7500 ± 1.5800b
Sucrose	Disaccharide	81.2667 ± 0.4933a	74.7667 ± 1.5373b	70.6198 ± 0.8892c
Maltose		0.0563 ± 0.0006b	0.0576 ± 0.0045b	0.0619 ± 0.0008a
Trehalose		0.0210 ± 0.0004c	0.0233 ± 0.0011a	0.0221 ± 0.0004b
Lactose		N	N	N
Inositol	Monosaccharide	12.3000 ± 0.5196b	16.6000 ± 0.1800a	10.5200 ± 1.0531c
D-Fructose		6.3767 ± 0.2802c	10.3333 ± 0.2517a	8.1347 ± 0.2435b
Glucose		4.1767 ± 0.1950c	9.7067 ± 0.2268a	5.3470 ± 0.8560b
D-Galactose		0.0774 ± 0.0019b	0.0821 ± 0.0015a	0.0736 ± 0.0019b
D-Arabinose		0.0455 ± 0.0007b	0.0468 ± 0.0010a	0.4780 ± 0.0017a
L-Fucose		0.0420 ± 0.0004b	0.0442 ± 0.0004a	0.0412 ± 0.0004b
L-Rhamnose		0.0278 ± 0.0005b	0.0308 ± 0.0006a	0.0289 ± 0.0005b
D- sorbose		0.0182 ± 0.0003a	0.0158 ± 0.0004b	0.0164 ± 0.0003b
Xylitol		0.0139 ± 0.0004a	0.0136 ± 0.0004a	0.0138 ± 0.0003a

The mean ± SD in three biological replicates was shown. Different letters indicated significant differences at *p* < 0.05. N: not detected

### Expression patterns of the known genes related to dormancy release after GA_3 _and GA_4 _treatments

**Fig. 7:**
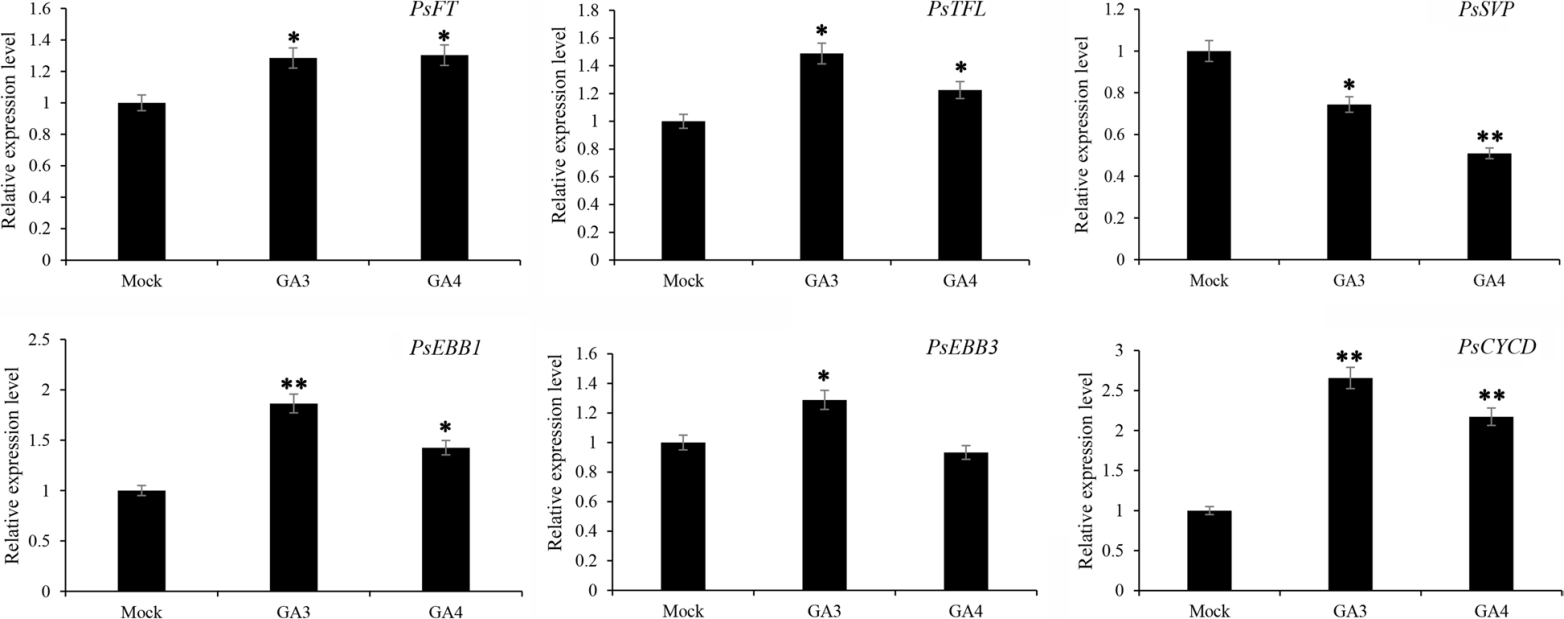
The relative expression levels of known dormancy-associated genes after GA_3_ and GA_4_ applications in *P. suffruticosa* cv ‘Luhehong’. Data were represented as mean of three different determinations ± SD. Asterisks indicate statistically significant differences (one-way ANOVA, *p < 0.05, **p < 0.01)

Based on the recent studies, the relative expression patterns of six genes including *PsFT*, *PsFTL, PsSVP, PsEBB1, PsEBB3, PsCYCD* were identified by qPCR (Fig. 7). *PsFT* showed significant increasing after both GA_3_ and GA_4_ feedings, and there was no significant difference between these two treatments, and *PsTFL* was upregulated by GA_3_ and GA_4_ treatments_._*PsSVP*, a MADS-box gene with homology to *SVP* in *Arabidopsis*, were significantly inhibited by GA_3_ and GA_4_ feedings, of which its expression level was down-regulated about 1.96-fold by GA_4_. *PsEBB1* and *PsEBB3*, encoding transcription factor of the AP2/ERF family, among them *PsEBB1* was significantly induced by GA_3_ and GA_4_ treatments, and *PsEBB3* showed significant upregulation only by GA_3_ feed. *PsCYCD*, encoding an important protein at cell cycle check point from G1 phase to S phase, quickly increased its expression level after GA_3_ and GA_4_ feedings, of which it was upregulated about 2.65-fold by GA_3_ treatment.

## Discussion

Bud dormancy transition is a vital developmental process for perennial plant survival and is precisely regulated by complex endogenous signaling networks, such as hormone signaling, sink/ source energy conversion, and multiple environmental signals, including low temperature and photoperiod. Tree peony is a well-known ornamental and medicinal plant with endodormancy characteristics. In recent years, the study on endodormancy regulation mechanisms became more and more urgent for the industrial development of anti-season culture. Practically, exogenous GA_3_ is a common method to assist dormancy release and bud burst in the anti-season production of tree peony [4, 39]. In some cases, such as Japanese apricot, GA_4_ treatment iswidely utilized to induce earlier bud break and sub-sequential growth [16]. Until now, the physiological effects of exogenous GA_3_ and GA_4_ on the dormancy release in tree peony were unknown. In this study, the morphological results showed that both GA_3_ and GA_4_ markedly accelerated dormancy release. However, the effect of GA_3_ was superior to GA_4_ in promoting bud dormancy release, following bud burst and subsequent growth in tree peony. In order to unlock the possible molecular mechanism of exogenous GAs accelerating bud endodormancy release, we performed a comprehensive transcriptome analysis of GA_3_ and GA_4_ treatments. Our results indicated that endogenous signals such as sugar metabolism, plant hormone signal transduction, including GA synthesis and signaling, ethylene, ABA, IAA, etc., cell cycle and photosynthesis played important roles in promoting bud endodormancy release and bud burst.

### Exogenous GAs applications regulate phytohormones metabolism and signaling

GAs are fundamental phytohormones that extensively regulate plant growth and development, especially bud dormancy and sprouting transition in perennial plants [16, 38, 40]. In tree peony, Mornya et al. considered that the accumulated GA was the key regulator to bud dormancy and growth [38]. Meanwhile, the expression profile of GA synthesis and signalling-releated genes and the up-regulated contents endogenous GAs during chilling-induced dormancy release, together suggested that GA pathway participated in the dormancy release in tree peony [41]. In this study, exogenous GA applications increased the contents of endogenous bioactive GAs including GA_1_, GA_3_, GA_4_ and GA_7_, of which GA_1_ and GA_3_ were more abundant in GA_3_-treated group than in GA_4_-treated group, while GA_4_ and GA_7_ were more in GA_4_ group (Fig. 5). The results suggested tree peony buds were more sensitive to GA_3_ considering the morphological effects of the two exogenous GAs, and exogenous GAs promoted dormancy release and subsequent bud development by the enhancement of endogenous bioactive GAs.

At the same time, the high levels of bioactive GAs feedback-regulate the expression of key GA synthesis enzyme genes, including *GA20ox*, *GA3ox*, *GA2ox*, etc. In tea, the transcripts of *CsGA3ox-2* and *CsGA20ox-1* were down-regulated, whereas *CsGA2ox* was up-regulated by exogenous GA_3_ treatment [14]. In addition, GA signaling transduction is also feedback-regulated by exogenous GA treatment [42]. Plant responds to GA by inducing the degradation of DELLAs, which depends on SCF E3 ubiquitin ligase complex recognizing and combining the GA-GID1-DELLA, and GID2 is an important subunit of E3 ubiquitin ligase [43]. In our results, DEGs were mainly enriched in plant hormone signal transduction after exogenous GAs applications, among which, *PsGA20ox* and *PsGID2* were down-regulated after GA_3_ and GA_4_ applications (Fig. 5). These results suggested exogenous GA_3_ and GA_4_ treatments promoted the interconversion of endogenous GAs, and the increasing contents of endogenous GAs feedback regulated the GA biosynthesis and signal transduction in tree peony.

ABA is another key hormone affecting dormancy release and bud break. The contents of ABA were down-regulated under spring temperature in tree peony [38]. ABA is responsible for the initiation and maintenance of endodormancy, as the increasing content of endogenous ABA presented at the onset of endodormancy, while the bud dormancy release is linked to declining ABA levels in several plants [44, 45]. Meanwhile, ABA catabolism by up-regulation of *VvA8H-CYP707A4*, an ABA 8’-hydroxylase, accelerated dormancy release of grapevine buds [10]. Therefore, ABA regulates endodormancy throughout its synthesis, metabolism and signalling process. In this study, several genes involving in ABA metabolic and signal transduction pathway were down-regulated after exogenous GA_3_ and GA_4_ treatments. For example, three ABA receptor genes *PsPYLs* and two ABA responsive element binding factor genes *PsABFs* decreased significantly after GAs applications, consistent with the decreasing contents of endogenous ABA (Fig. 5). It was speculated that exogenous GAs accelerated dormancy release partially by inhibiting endogenous ABA synthesis and signaling.

It is believed that Dormancy-associated MADS-box (DAM) functions downstream of the ABA signalling pathway to regulate bud dormancy. Recent study found that pear ABRE-BINDING FACTOR3 (PpyABF3) directly bound to the *PpyDAM3* promoter and positively enhance its expression [9]. In poplar, SVL, a DAM homolog, negatively regulates GA pathway but positively accelerates ABA to induce and maintain dormancy, and prolonged chilling depresses *SVL* expression and induces *FT1* and GA biosynthesis [23, 28]. In our study, *PsABFs* and *PsSVP* significant decreased after GAs feedings, and the down-regulation of *PsABFs* in GAs treated buds suggested that GAs might also adjust dormancy through a DAM pathway, and there might be a loop circuit between GA and ABA in dormancy regulation of tree peony.

Auxin is an important hormone involving in many plant developmental processes. In *Pinus Sylvestris*, an increase of IAA level was induced by exogenous GA_4_ in the cambial region, resulting in stimulation of tracheid production in the terminal shoot of intact plants. Similarly, the IAA content and the related genes were up-regulated from the dormancy to the reactive stage in *Prunus mume* [46]. Besides the increase of auxin content, auxin-signaling pathways were also enriched in the vascular cambium during the transition from dormancy to active growth in Chinese fir [47]. Here, two DEGs encoding indole-3-acetic acid-amido synthetase GH3 family members, *PsGH3s*, were significantly up-regulated after GA_3_ and GA_4_ treatments, which was consistent with the previous results in *Prunus mume* [46]. The contents of endogenous IAA significantly increased only by exogenous GA_3_ application, which was inconsistent with the expression level of *PsGH3* (Fig. 5). We suspected that GA_4_ could also enhance the increasement of endogenous IAA, but later than GA_3_ feeding in chronological order, which partially contributed to the morphological effect variation of different GAs.

### Activation of carbohydrate metabolism involves in GAs-induced dormancy release

Carbohydrates not only act as an energy source but also as precursors for metabolic processes, and some of hexoses as molecular signals regulating many vital steps. Dormant buds of perennial woody plants seem to be in an inactive state with limited metabolic activities, and the reconfiguration of carbohydrate metabolism is related to the transition process from dormancy to regrowth [26, 46]. Our recent study indicated that carbohydrate metabolism, particularly the activation of PPP pathway, might play an important role in chilling-induced dormancy release of tree peony [33]. In this study, the significant degradation of starch and dramatic changes in soluble sugars emerged during GA-induced dormancy release in tree peony. Accompanied by the decreasing of starch level, the contents of soluble glucose and fructose showed up-regulated pattern after GA_3_ and GA_4_ applications, which were consistent with the expression levels of key gene involved in starch metabolism, sucrose transporter and glucose biosynthesis, including *PsBMY*, *PsAMY*, *PsTPS* and *PsBGL* (Fig. 6 and Table 3). Therefore, vast carbohydrates undoubtedly served as a strong energy sink for bud burst and subsequent growth after exogenous GAs applications in tree peony. These results agreed with those of leafy spurge, grape and *Prunus mume* crown buds during the dormancy-activity transition process [46, 48, 49]. Together, that exogenous GAs accelerated bud dormancy release and the following growth was associated with the induction of soluble sugar increase to satisfy the energy and substance requirement for bud regrowth.

The crosstalk between sugar and hormones including GA, ABA and IAA had been reported. The α-amylases act as key enzymes in starch degradation to generate soluble sugars, which play critical roles in the dormancy process and are tightly regulated by GA. For example, during the bud dormancy process in leafy spurge, GA promoted the synthesis and increased activity levels of α-amylases, suggesting GA might involve in the breakdown of starch. In grape, exogenous GA_3_ induced the expression of *Vva-AMY3* and *Vva-AMY4* during the GA_3_-induced dormancy release of grapevine bud [49]. In-depth, GA promoted the production of α-amylases mediating by *GAMYB* [50], while the induction of α-amylase triggered by GA was repressed by sugars, and this repression was transduced by a pathway independent of gibberellin signaling or that repression occurred at the level of *GAMYB* translation [51]. In general, ABA repressed the production of α-amylases by an ABA-induced protein kinase (PKABA1) and two ABA-induced WRKY [52, 53], and GA induction effect of the α-amylase gene was repressed by ectopic expression of wild-type ABA responsive element binding factor (ABF) 1 and 2 [54]. In this study, the down-regulation ABA might result in the increasing expression of α-amylase after exogenous GAs appication, which indicated the important role of interaction between sugar and hormones during GA-induced dormancy release in tree peony.

On the other hand, sugars also function as signaling molecules in plant growth regulations and stress responses. Strong evidences show that sugar signaling molecules, including sucrose, glucose, fructose and trehalose-6-phosphate, regulate development either directly or through interactions with other signaling pathways such as hormone-mediated processes [55]. For example, Glucose increase can activate target of rapamycin (TOR) kinase signaling and modulate indole-3-acetic acid (IAA) biosynthesis and transport, which will activate the meristem, promote cell division and expansion, and hence promote growth and development [56]. Trehalose-6-phosphate also plays crucial signaling roles in stimulating plant growth and development through repression of SnRK1 [57]. Our results revealed that GAs application promoted the degradation of sucrose and thus produced more glucose, fructose and trehalose-6-phosphate, accompanied by higher levels of IAA, and cell division was accelerated according to the expressions of related genes and the morphological changes (Fig. 1, 5 and 6). The results indicated that sucrose, glucose, fructose and trehalose-6-phosphate might function as sugar signaling molecules in GAs induced dormancy release, and GAs application might boost sugar signaling transduction.

As well known that photosynthesis ensures the continuous energy supply in the higher plant, which is the prerequisite for further growth and development. Our result indicated that exogenous GAs treatments increased the photosynthetic rate, while stomatal conductance and transpiration rate significantly enhanced only by GA_3_ in leaves, which well explained the morphological divergences after GAs treatments, similar to the effect of GA_3_ in the tree peony bracts [58]. FNR proteins existing in photosynthetic tissues is mainly responsible for catalyzing the final reaction of linear electron transport in photosynthesis, catalyzing electron from reduced ferredoxin (Fd) to NADP^+^, and the NADPH is mainly used for CO_2_ fixation and other metabolic processes of chloroplasts in the Calvin cycle [59]. In our study, the transcript of *PsFNR* significantly increased after GAs treatments, and its increased expression level by GA_3_ was more than by GA_4_, which suggested that GA_3_ promoting the growth of shoots better than GA_4_ might because that GA_3_ feeding accelerated photosynthesis to generate more energy substances.

### GAs treatments accelerate cell cycle and promote the bud growth

Both GA_3_ and GA_4_ significantly promoted dormancy release and branch elongation. The increase of cell number is a prerequisite for the elongation of branches, which means more cells are in the Mitosis phase, and chromatin condensing is an important characteristic of Mitotic phase cell. The condensing complex promotes chromosome condensation during mitosis, and Structural Maintenance of Chromosomes 4 (SMC4) and SMC2 are the core components [60]. The abundance of SMC4 fluctuated with cell cycle and peaked at Mitotic stage and fall at interphase in budding yeast. In our study, *PsSMC4* significantly increased after GAs treatments, which implied that more cell entered into Mitotic phase after GAs applications. The cyclin A/cdc2-kinase regulates the transition from G2 to Mitotic phase, and cyclin A (CYCA) is an important regulatory subunit. Here, the abundance of *PsCYCA* significantly increased by GA_3_, which might promote mores cells to enter Mitotic phase after GA_3_ treatment, which will accelerate bud burst and shoot growth. Whereas there was no difference between GA_4_ and mock. Additionally, the heterohexameric minichromosome maintenance complex (MCM2-7) serves as the central DNA replicative helicase in eukaryotes [61]. Two *PsMCM2* and one *PsMCM3* significantly decreased after GA_3_ treatment but increased by GA_4_ feedings, which were mainly consistent with the expression pattern of *PsCYCA* (Fig. 6), suggesting the condensed structure of chromosome in Mitotic phase after GA_3_ treatment.

### The putative reasons for GA_3_ superior to GA_4_ promoting bud dormancy release

There are four major endogenous bioactive GAs, i.e. GA_1_, GA_3_, GA_4_ and GA_7_ in most plant species. In practice, exogenous GA_3_ is widely applied in dormancy breaking, such as in *Paeonia lactiflora* [17], *Rhododendron simsii* Planch [15], *Camellia sinensis* [62], *Prunus armeniaca* L. [63], etc. In some cases, GA_4_ is applied and more effectively to induce dormancy release and bud burst in hybrid aspen and Japanese apricot [35, 64]. In hybrid aspen, exogenous GA_4_ induces canonical bud burst and shoot elongation, but GA_3_ application only generates callus-like tissue. In contrast, GA_3_ and GA_4_ treatments exhibited similar morphological changes in tree peony, but GA_3_ induced faster bud burst, longer shoot and higher flowering rate in tree peony (Fig. 1), indicating GA_3_ was superior to GA_4_ in tree peony forcing culture.

On mechanism, Rinne et al. found GA_3_ and GA_4_ up-regulated different 1,3-β-glucanase members (glucan hudrolase family 17, GH17) in *Populus* that could hydrolyze 1,3-β-glucan deposited at pores and plasmodesmata in dormant stage. However, GA_4_ application could thoroughly reopen the signal conduit but GA_3_ was less efficient [35]. We found that both GA_3_ and GA_4_ induced same *GH17* members but in diverse degrees in tree peony, which was in depth with the morphological variations (unpublished data). Therefore, plants do not response to phytohormone in a common manner.

**Fig. 8:**
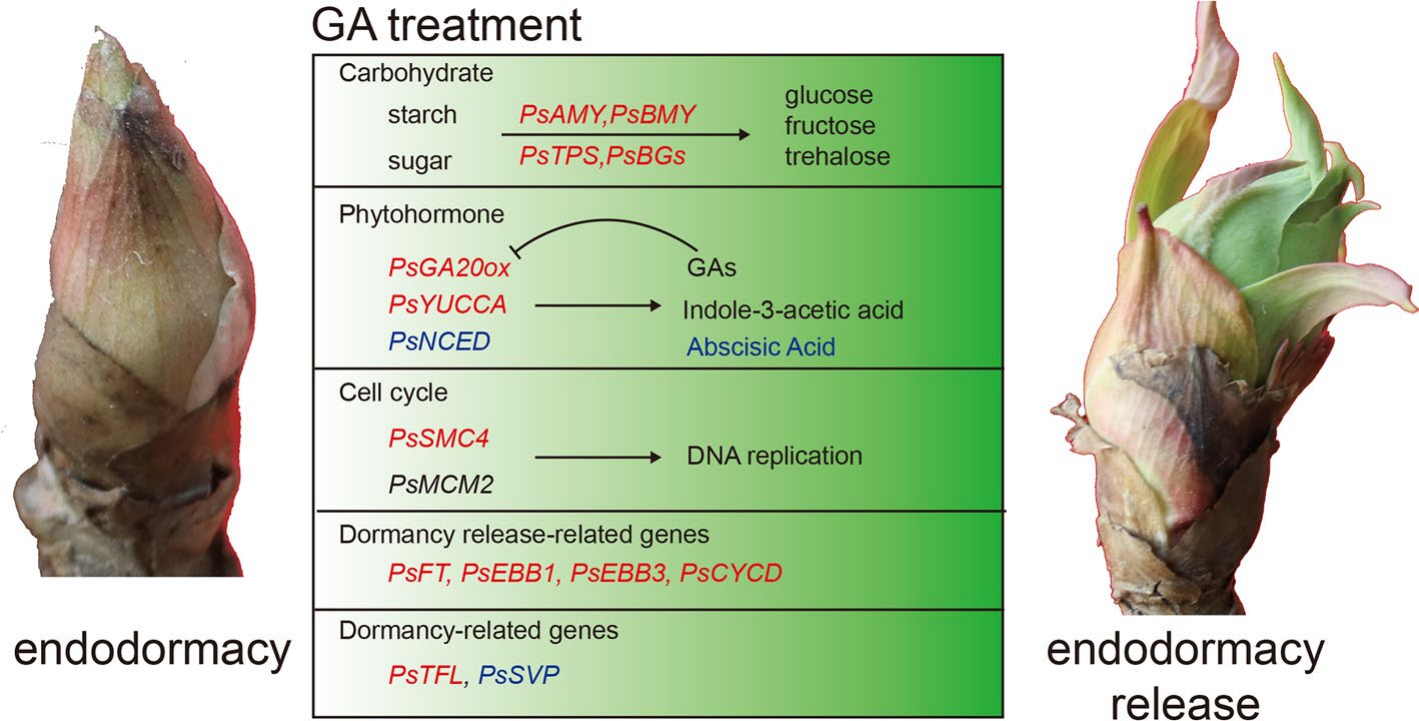
Model of the putative mechanism about exogenous GA_3_ and GA_4_ on dormancy release in tree peony. Exogenous GA_3_ and GA_4_ promote dormancy release and subsequent growth by increasing endogenous bioactive GAs (GA_1_, GA_3_, GA_4_ and GA_7_) and IAA, decreasing ABA contents, enhancing the contents of soluble glucose and accelerating cell division, promoting the expression levels of known four dormancy release-related genes including *PsFT*, *PsEBB1*, *PsEBB3*, *PsCYCD* and one dormancy-related gene *PsTFL,* inhibiting that of *PsSVP*. Red represents the up-regulated gene and blue represents the down-regulated gene

Our results indicated that exogenous GA_3_ was superior to GA_4_ in accelerating dormancy release and subsequent growth (Fig. 1), despite more DEGs were screened in GA_4_ vs mock group. Collectively, we concluded several possible reasons. Firstly, the contents of endogenous GAs and the expression patterns of the related-genes including *PsGA20ox* and *PsGID2* suggested that tree peony was more sensitive to exogenous GA_3_ than to GA_4_, and GA_3_ treatment enhanced more endogenous bioactive GA_1_ and GA_3_, which might function more effectively in tree peony buds. Secondly, *PsBMY* was only induced by GA_3_ treatment, and the higher expressions of *PsAMY* and *PsTPS* were up-regulated after GA_3_ feeding, which lead to the more effective hydrolysis of starch to offer more energy for break dormancy, and photosynthesis of leaves by GA_3_ application ensured the continuous energy supply. The quantification of starch and sugar further proved this speculation, and higher phtosythetic ability achieved after GA_3_ feeding accompanied by higher *PsFNR* expression. Thirdly, GA_3_ application resulted in higher IAA levels, another growth enhancer hormone. In addition, the interactions between sugar and hormones were also an important factor during GA-induced dormancy release, and GA_3_ induced more effectively sugar signaling transduction to stimulate cell division. The expression patterns of the cell cycle and DNA replication related genes, including *PsCYCA*, *PsCYCD*, *PsSMC4*, *PsMCM2* and *PsMCM3,* suggested that exogenous GA_3_ feeding more effectively accelerated cell cycle to promote dormancy release and subsequent growth (Fig. 8). At last, we evaluated the expressions of several dormancy-associated genes, such as dormancy release-related *FT*, *EBB1*, *EBB3* and *CYCD*, and dormancy-associated *TFL* and *SVP*. Higher expression levels were detected by GA_3_ treatment for *TFL*, *FT*, *EBB1*, *EBB3* and *CYCD*, but lower for *SVP*, implying more efficiency of GA_3_.

## Conclusions

High-quality Full-length transcriptome was obtained in dormant buds of tree peony, and was used to analyze the effects of GAs on bud dormancy and growth. Morphologically, exogenous GA_3_ and GA_4_ accelerated bud dormancy release, subsequent growth and flowering, and the intrinsic mechanism might be associated with the increasing endogenous bioactive GAs (GA_1_, GA_3_, GA_4_ and GA_7_) and IAA, decreasing ABA contents, enhancing the contents of soluble glucose, fructose and trehalose to provide sufficient energy and metabolic substance, and accelerating sugar signaling and cell division. The reason of GA_3_ superior to GA_4_ treatment might be that tree peony was more sensitive to GA_3_ than to GA_4_, and GA_3_ application might be more efficient to accelerate cell cycle and starch hydrolysis to provide more soluble sugar as energy and carbon skeleton for other metabolites synthesis along with stepped-up sugar signaling. All results would provide valuable data for the mechanism of dormancy release and the foundation for forcing culture improvement in tree peony.

## Materials and methods

### Plant materials and morphological observation

Tree peony (*Paeonia suffruticosa*) ‘Luhehong’ was formally identified by Heze Zhaolou Tree-peony Garden in 1968. It is a traditional cultivar and not protected by plant variety rights in China. Therefore, no specimen of this material is deposited in a publicly available herbarium. We have obtained the permission of Tree Peony Research Institute of Qingdao Agricultural University (Qingdao, China) to collect tree peony ‘Luhehong’. Four-year-old ‘Luhehong’ were potted and treated in cold conditions (0–4 °C, 24 h-dark) from Nov 10, 2018. The apical buds after 0, 7, 14, 21, 28 d chilling applications were collected and immediately frozen in liquid nitrogen, which were stored at -80 °C until use for PacBio full-length transcriptome sequencing, and three replicates (3 plants/replicate) per treatment were set, respectively. On Nov 17, 2018, 27 plants were transferred to a greenhouse (25 °C, 8-h-light/16-h-dark), and then GA_3_ (500 mgּּּ L^−1^) and GA_4_ (500 mgּ L^−1^) were applied on apical buds, respectively, the samples treated by sterile distilled water was used as mock. After treated for 48 h, the apical buds were harvested, immediately frozen in liquid nitrogen and stored at -80 °C until use for Illumina transcriptome sequencing, qPCR, sugar and starch evaluation, and endogenous phytohormone measurement, respectively. Three replicates (3 plants/replicate) per group were set.

Morphological observation was conducted on another 27 plants. Sprouting rate was observed every day till GA_3_ and GA_4_ applications for 16 d [18]. Branch height, the first leaf length and flowering rate were monitored and measured as described previously [5]. Treatment effects were analyzed using ANOVA followed by Duncan's multiple range tests at a significance level of 0.05, and the *p*-value of the comparison with the treated group and Mock was obtained after t-test by SPSS 13.0 for Windows (SPSS, USA).

### Measurement of photosynthesis

The net photosynthetic rate (Pn), intercellular CO_2_ concentration (Ci), stomatic conductance (Gs), and transpiration rate (Tr) were evaluated 20 d after GAs applied, by a CIRAS-3 portable photosynthesis system (PP Systems, USA) under ambient CO_2_ concentrations at 20 °C, 1200 μmol (photon) m^−2^ s^−1^ light, and 80% relative humidity. The first leaf under flower was measured and 10 pieces of leaves per repeat.

### Transcriptome sequencing and analysis

Ten buds were sampled per biological replicate to produce an independent pool. PacBio full-length sequencing and Illumina sequencing were both performed by OE Biotech Co. Ltd. (Shanghai, China). Total RNA was extracted using RNA extracting kit (Umagen, China) according to the manual, and the DNA contaminates were excluded using TURBO DNA-free Kit (Thermo, USA). RNA samples were checked on a 1.5% (*w*/*v*) agarose gel, and the integrities were evaluated using the Agilent 2100 Bioanalyzer (Agilent Technologies, Santa Clara, CA, USA). The samples with RNA Integrity Number (RIN) ≥ 8.5, OD_260_/OD_280_: 2.0 ~ 2.2, OD_260_/OD_230_: 1.8 ~ 2.1, qubit/nano: 0.5 ~ 2.0 were subjected to the subsequent analysis.

### PacBio full-length sequencing of mixed samples

In order to generate a representatively transcriptomic reference, 1 μg of total RNA extracted from each chilling-treated sample were equally pooled together and used for cDNA synthesis using SMARTer PCR cDNA Synthesis Kit (Clontech, USA). The library construction and PacBio sequencing were performed according to the official protocol as described by Pacific Biosciences (Pacific Biosciences, USA). Briefly, a total of 12 PCR cycles of amplification were performed using PrimeSTAR GXL DNA Polymerase (Clontech, USA). After purification with AMPure PB Beads, the cDNA products were then subjected to the construction of SMRTbell template libraries using the SMRTbell Template Prep Kit 1.0 (Pacific Biosciences, USA). Finally, SMRT cells were sequenced on a PacBio Sequel instrument using sequencing kit 2.1 with 10 h movie recordings. Sequencing reads were subjected to Circular Consensus Sequences (CCS) using the SMRT Analysis Software (https://www.pacb.com/products-and-services/analytical-software/devnet/).

### Illumina sequencing of GA_3_, GA_4 _and mock group

The libraries were constructed using TruSeq Stranded mRNA LTSample Prep Kit (Illumina, San Diego, CA, USA) according to the manufacturer’s instructions. Then these libraries were sequenced on the Illumina sequencing platform (HiSeqTM 2500). The raw reads obtained by Illumina Hiseq 2500 sequencer were originally trimmed by removing the adapter sequences, and then the low-quality sequences with quality scores of less than 20 and the short reads with lengths of less than 10 bp were also removed. Finally, the high-quality clean reads were obtained for transcriptome de novo assembly using Trinity (version 20,140,717) [65], and redundant sequences were filtered using TGICL [66].

### Unigenes annotation and transcriptome analysis

PacBio reads were classified into full-length and non-full-length sequences, and then reads were corrected using LoRDEC. The redundant sequences were removed using CD-HIT. The merged unigenes were annotated based on sequence similarity using BLASTx and BLASTn algorithms against the NCBI non-redundant database, and only significant BLAST results (E-value < 1e-5) were considered to be annotated, including protein functional annotation, COG functional annotation, GO functional annotation, and pathway annotation. Then, the best alignment results were used to determine the sequence direction and protein-coding-region prediction (CDS) of the unigenes. The functional category was assigned to each gene product according to the gene ontology (GO) terms (www.geneontology.org) that were analyzed using Blast2GO software (http://www.blast2go.com/b2ghome). Pathway assignment was performed according to the Arabidopsis database classification in the KEGG database (Kyoto Encyclopedia of Genes and Genomes, http://www.genome.jp/kegg). Based on the KEGG database, the complex biological behavior of the genes was analyzed through pathway annotation. Up to now, there is still no high-quality genome data in tree peony, and the clean reads were mapped to our PacBio full-length transcriptome using the short oligonucleotide analysis package SOAPaligner/soap2 (version 2.21).

### Identification of differentially expressed genes

The expression levels of unigenes were generally evaluated by the quantity of fragments per kilobase of exon model per million mapped fragments (FPKM). False discovery rate (FDR) was used to correct for *p*-value thresholds in multiple testing [67]. After comparing each other using EDGER software (empirical analysis of digital gene expression data in R) [68], the unigenes with |log2Ratio|≥ 1 and *p* < 0.05 were identified as differentially expressed genes (DEGs). Hierarchical cluster analysis of DEGs was performed to explore gene expression patterns. Then, DEGs were subjected to GO functional enrichment and KEGG pathway enrichment analyses. GO terms and KEGG pathways fulfilling the criterion of a Bonferroni-corrected *p*-value ≤ 0.05 were defined as significantly enriched in DEGs.

### Real-time quantitative RT-PCR

In order to verify the sequencing quality and the expression patterns of DEGs, the associated primer pairs were designed based on their partial cDNA sequences (Additional file 8)*.* Two mg total RNA was used to synthesize the first-strand cDNA using the PrimerScript™ RT reagent Kit according to the manufacturer’s instructions (TaKaRa, Dalian, China), and then the cDNAs were three times diluted as qPCR template. PCR reaction was performed in 20 mL reaction mixture (2 × SYBR Green Master mix of 10 mL, 10 mmolּ L^−1^ each primer of 1 mL, 3 × diluted cDNA template 2 mL and 6 mL ddH_2_O) using SYBR® Premix Ex Taq™ II Kit (TaKaRa, Dalian, China). The PCR reactions were run in a LightCycler® 480 II (Roche, USA), and the program was 95 °C for 2 min, and followed by 40 cycles of 95 °C for 10 s, 55 °C for 30 s and 72 °C for 30 s. All reactions were conducted in triplicates for each sample, and the relative expression levels were calculated using the method of 2^−△△Ct^ [69], *PsActin* was used as the reference gene. Significance was tested using SPSS 13.0 for Windows (SPSS, USA).

### Measurement of endogenous phytohormone

The endogenous phytohormone contents of three groups (mock, GA_3_ and GA_4_) were determined by Wuhan Greensword Creation Technology Company (http://www.greenswordcreation.com) based on LC–MS/MS analysis according to a previously reported method [70].

### Determination of the sugar and starch content

The freeze-dried samples (mock, GA_3_ and GA_4_) were crushed using a mixer mill (MM 400, Retsch) with a zirconia bead for 1.5 min at 30 Hz. Twenty mg of powder was diluted to a 500 μL methanol: isopropanol: water (3:3:2, V/V/V), vortexed for 3 min and ultrasound for 30 min. The extract was centrifuged at 14 000 rpm for 3 min (4 °C). The supernatants were collected for 50 μL and evaporated under nitrogen gas stream, and add internal standard. The extract was evaporated under nitrogen gas stream and transferred to the lyophilizer for freeze-drying. The residue was used for further derivatization as follows: the sample of small molecular carbohydrates was mixed with 100 μL solution of methoxyamine hydrochloride in pyridine (15 mg·mL^−1^). The mixture was incubated at 37 °C for 2 h. Then 100 μL of BSTFA was added into the mixture and kept at 37 °C for 30 min after vortex-mixing. Dilute the mixture to the appropriate concentration with n-hexane. The mixture was analyzed by GC–MS/MS (Agilent 7890B-7000D) according to Sun et al. [71]. The starch content was measured according to the previous method [72, 73]. Three replicates of each assay were performed.

## Supplementary Information

**Additional file 1:** The statistical data of unigenes annotated in public database.

**Additional file 2:** The number distribution of the unigenes encoding transcription factor (TF).

**Additional file 3:** PCA analysis of the samples.

**Additional file 4:** GO analysis of DEGs (*p* value<0.05 & |log_2_FC|>1) after GA_3_ and GA_4_ applications.

**Additional file 5:** The transcriptomic data of the overlapping 849 DEGs between GA_3_ vs GA_4_ and GA_3_ vs mock, GA_3_ vs GA_4_ and GA_4_ vs mock, respectively.

**Additional file 6:** Based on Neighbor-Joining (NJ) model by MEGA 6.0, the phylogenetic trees of the related DEGs enriched in the KEGG pathways about plant hormone signal transduction, starch and sucrose metabolism, cell division and DNA replication. Genetic distance was calculated based on nucleotide difference (p-distance) with complete deletion of gaps. Scale bar = 0.05. The number at each node indicates the percentage of bootstrapping of 1000 replications.

**Additional file 7:** The correlation between the DEGs fold change of expression levels by qRT-PCR and the corresponding fold change with RNAseq.

**Additional file 8:** Primer sequences used for qPCR analysis in this study.

## Data Availability

The transcriptome data of the overlapping 849 DEGs between GA_3_ vs GA_4_ and GA_3_ vs mock, GA_3_ vs GA_4_ and GA_4_ vs mock have been appended as Additional file 5. The full-length transcriptome data have been submitted to the public repository (SRA), and the BioProject ID is PRJNA720276. The RNAseq data used during the current study available from the corresponding author (Gai Shupeng: spgai@qau.edu.cn) on reasonable request.

## Abbreviations

ANOVA: Analysis of variance
cDNA: Complementary DNA
COG: Clusters of Orthologous Groups
GC–MS/MS: Gas chromatography-mass spectrometry
GO: Gene Ontology
IAA: Indole-3-acetic acid
LC–MS/MS: Liquid chromatography electrospray ionisation tandem mass spectrometry
PCA: Principal component analysis

## References

[1] Lang GA, Early JD, Martin GC, Darnell RL (1987). Endo-, para-, and ecodormancy: physiological terminology and classification for dormancy research. Hortscience.

[2] Heide OM, Prestrud AK (2005). Low temperature, but not photoperiod, controls growth cessation and dormancy induction and release in apple and pear. Tree Physiol.

[3] Carvalho RIND, Biasi LA, Zanette F, Rendoke JC, Santos JM, Pereira GP. Dormancy of “Imperial Gala” apple and “Hosui” pear tree buds in a region of low chill occurrence. Acta Sci Agron. 2014;36(4):429–34.

[4] Zhang T, Si F, Zhang Y, Gai S (2018). Effect of exogenous gibberellin on DNA methylation level and expression of related enzyme genes in tree peony floral buds. Scientia Agricultura Sinica.

[5] Zhang Y, Si F, Wang Y, Liu C, Zhang T, Yuan Y, Gai S (2020). Application of 5-azacytidine induces DNA hypomethylation and accelerates dormancy release in buds of tree peony. Plant Physiol Bioch.

[6] Hoeberichts FA, Povero G, Ibañez M, Strijker A, Pezzolato D, Mills R, Piaggesi A (2017). Next Generation Sequencing to characterise the breaking of bud dormancy using a natural biostimulant in kiwifruit (*Actinidia deliciosa*). Sci Hortic-Amsterdam.

[7] Michailidis M, Karagiannis E, Tanou G, Sarrou E, Adamakis ID, Karamanoli K, Martens S, Molassiotis A (2018). Metabolic mechanisms underpinning vegetative bud dormancy release and shoot development in sweet cherry. Environ Exp Bot.

[8] Wu R, Tomes S, Karunairetnam S, Tustin SD, Hellens RP, Allan AC, Macknight RC, Varkonyigasic E (2017). SVP-like MADS box genes control dormancy and budbreak in Apple. Front Plant Sci.

[9] Yang Q, Yang B, Li J, Wang Y, Tao R, Yang F, Wu X, Yan X, Ahmad M, Shen J, Bai S, Teng Y (2020). ABA-responsive ABRE-BINDING FACTOR3 activates DAM3 expression to promote bud dormancy in Asian pear. Plant Cell Environ.

[10] Zheng C, Acheampong AK, Shi Z, Mugzech A, Halalybasha T, Shaya F, Sun Y, Colova V, Mosquna A, Ophir R (2018). Abscisic acid catabolism enhances dormancy release of grapevine buds. Plant Cell Environ.

[11] Huang X, Xue T, Dai S, Gai S, Zheng C, Zheng G (2008). Genes associated with the release of dormant buds in tree peonies (*Paeonia suffruticosa*). Acta Physiol Plant.

[12] Cooke JEK, Eriksson ME, Junttila O (2012). The dynamic nature of bud dormancy in trees: environmental control and molecular mechanisms. Plant Cell Environ.

[13] Olsen JE (2010). Light and temperature sensing and signaling in induction of bud dormancy in woody plants. Plant Mol Biol.

[14] Yue C, Cao H, Hao X, Zeng J, Qian W, Guo Y, Ye N, Yang Y, Wang X (2018). Differential expression of gibberellin- and abscisic acid-related genes implies their roles in the bud activity-dormancy transition of tea plants. Plant Cell Rep.

[15] Nell TA, Larson RA (1974). The influence of foliar applications of GA_3_ GA_4_,_7_ and PBA on breaking flower bud dormancy on azalea CVS redwing and dogwood. J Horticult Sci.

[16] Zhuang W, Gao Z, Wang L, Zhong W, Ni Z, Zhang Z (2013). Comparative proteomic and transcriptomic approaches to address the active role of GA_4_ in Japanese apricot flower bud dormancy release. J Exp Bot.

[17] Evans MR, Anderson NO, Wilkins HF (1990). Temperature and GA_3_ effects on emergence and flowering of potted *Paeonia lactiflora*. Hortsci Publ Am Soc Horticult Sci.

[18] Gai S, Zhang Y, Liu C, Zhang Y, Zheng G (2013). Transcript profiling of *Paoenia ostii* during artificial chilling induced dormancy release identifies activation of GA pathway and carbohydrate metabolism. Plos One.

[19] Middleton AM, Úbeda-Tomás S, Griffiths J, Holman T, Hedden P, Thomas SG, Phillips AL, Holdsworth MJ, Bennett MJ, King JR, Owen MR (2012). Mathematical modeling elucidates the role of transcriptional feedback in gibberellin signaling. Proc Natl Acad Sci USA.

[20] Hedden P (2002). Gibberellin metabolism and its regulation. J Plant Growth Regul.

[21] Lin L, Huo X, Wen L, Gao Z, Muhammad KUR (2018). Isolation and role of *PmRGL2* in GA-mediated floral bud dormancy release in Japanese apricot (*Prunus mume* Siebold et Zucc.). Front Plant Sci.

[22] Azeez A, Zhao YC, Singh RK, Yordano VYS, Dash M, Miskolczi P, Stojkovi K, Strauss SH, Bhalerao RP, Busov VB (2021). EARLY BUD-BREAK 1 and EARLY BUD-BREAK 3 control resumption of poplar growth after winter dormancy. Nat Commun.

[23] Singh RK, Miskolczi P, Maurya JP, Bhalerao RP (2019). A tree ortholog of SHORT VEGETATIVE PHASE floral repressor mediates photoperiodic control of bud dormancy. Curr Biol.

[24] Tylewicz S, Petterle A, Marttila S, Miskolczi P, Azeez A, Singh RK, Immanen J, Mahler N, Hvidsten TR, Eklund DM (2018). Photoperiodic control of seasonal growth is mediated by ABA acting on cell-cell communication. Science.

[25] Yang Q, Niu Q, Tang Y, Ma Y, Yan X, Li J, Tian J, Bai S, Teng Y. PpyGAST1 is potentially involved in bud dormancy release by integrating the GA biosynthesis and ABA signaling in “Suli” pear (*Pyrus pyrifolia* White Pear Group). Environ Exp Bot. 2019;162:302–12.

[26] Ruttink T, Arend M, Morreel K, Storme V, Rombauts S, Fromm J, Bhalerao RP, Boerjan W, Rohde A (2007). A molecular timetable for apical bud formation and dormancy induction in poplar. Plant Cell.

[27] Aleman F, Yazaki J, Lee M, Takahashi Y, Kim AY, Li Z, Kinoshita T, Ecker JR, Schroeder JI (2016). An ABA-increased interaction of the PYL6 ABA receptor with MYC2 transcription factor: A putative link of ABA and JA signaling. Sci Rep-Uk.

[28] Singh RK, Maurya JP, Azeez A, Miskolczi P, Tylewicz S, Stojkovič K, Delhomme N, Busov V, Bhalerao RP (2018). A genetic network mediating the control of bud break in hybrid aspen. Nat Commun.

[29] Bonicel A, Verçosa DMRN (1990). Variation of starch and soluble sugars in selected sections of poplar buds during dormancy and post-dormancy. Plant Physiol Bioch.

[30] Ito A, Sakamoto D, Moriguchi T (2012). Carbohydrate metabolism and its possible roles in endodormancy transition in Japanese pear. Sci Hortic-Amsterdam.

[31] Jin F, Li J, Ding Q, Wang Q, He X (2017). Proteomic analysis provides insights into changes in the central metabolism of the cambium during dormancy release in poplar. J Plant Physiol.

[32] Wang W, Su X, Tian Z, Liu Y, Zhou Y, He M (2018). Transcriptome profiling provides insights into dormancy release during cold storage of *Lilium pumilum*. BMC Genomics.

[33] Zhang Y, Yu D, Liu C, Gai S (2018). Dynamic of carbohydrate metabolism and the related genes highlights PPP pathway activation during chilling induced bud dormancy release in tree peony (*Paeonia suffruticosa*). Sci Hortic-Amsterdam.

[34] Noriega X, Pérez FJ (2017). Cell cycle genes are activated earlier than respiratory genes during release of grapevine buds from endodormancy. Plant Signal Behavior.

[35] Rinne PLH, Schoot CVD (2011). Chilling of dormant buds hyperinduces FLOWERING LOCUS T and recruits GA-Inducible 1,3-β-glucanases to reopen signal conduits and release dormancy in *Populus*. Plant Cell.

[36] Rozi M, Chieh-Ting W, Cathleen M, Olga S, Dye SJ, Puzey JR, Elizabeth E, Xiaoyan S, Richard M, Strauss SH (2010). *Populus* CEN/TFL1 regulates first onset of flowering, axillary meristem identity and dormancy release in *Populus*. Plant J.

[37] Yordanov YS, Ma C, Strauss SH, Busov VB (2014). EARLY BUD-BREAK 1 (EBB1) is a regulator of release from seasonal dormancy in poplar trees. Proc Natl Acad Sci USA.

[38] Mornya PMP, Cheng F (2013). Seasonal changes in endogenous hormone and sugar contents during bud dormancy in tree peony. J Appl Hortic.

[39] Guan Y, Xue J, Xue Y, Yang R, Wang S, Zhang X (2019). Effect of exogenous GA_3_ on flowering quality, endogenous hormones, and hormone- and flowering-associated gene expression in forcing-cultured tree peony (*Paeonia suffruticosa*). J Integr Agr.

[40] Schrader J, Moyle R, Bhalerao R, Hertzberg M, Lundeberg J, Nilsson P, Bhalerao RP (2004). Cambial meristem dormancy in trees involves extensive remodelling of the transcriptome: Cambial meristem dormancy. Plant J.

[41] Zhang Y, Zhang W, Li Y, Liu C, Zheng G, Gai S (2014). The study of *PsGA20*ox gene participating in endo-dormancy release of flower buds by chilling treatment in tree peony. Acta Agriculturae Boreali-Sinica.

[42] Li M, An F, Li W, Ma M, Feng Y, Zhang X, Guo H (2016). DELLA proteins interact with FLC to repress flowering transition. J Integr Plant Biol.

[43] O’Neill DP, Davidson SE, Clarke VC, Yamauchi Y, Yamaguchi S, Kamiya Y, Reid JB, Ross JJ. Regulation of the gibberellin pathway by auxin and DELLA proteins. Planta. 2010;232(5):1141–9. 10.1007/s00425-010-1248-0 20706734

[44] Berruti A, Christiaens A, Scariot V, Van LMC (2012). Changes in ABA levels in vegetative and flower buds during dormancy in *Camellia*. Acta Hort.

[45] Xu R, Niimi Y, Han D (2006). Changes in endogenous abscisic acid and soluble sugars levels during dormancy-release in bulbs of *Lilium rubellum*. Sci Hortic-Amsterdam.

[46] Zhang Z, Zhuo XK, Zhao K, Zheng T, Zhang Q (2018). Transcriptome profiles reveal the crucial roles of hormone and sugar in the bud dormancy of *Prunus mume*. Sci Rep-Uk.

[47] Qiu Z, Wan L, Chen T, Wan Y, He X, Lu S, Wang Y, Lin J (2013). The regulation of cambial activity in Chinese fir (*Cunninghamia lanceolata*) involves extensive transcriptome remodeling. New Phytol.

[48] Anderson JV, Gesch RW, Jia Y, Chao WS, Horvath DP (2005). Seasonal shifts in dormancy status, carbohydrate metabolism, and related gene expression in crown buds of leafy spurge. Plant, Cell Environ.

[49] Rubio S, Donoso A, Pérez FJ. The dormancy-breaking stimuli “chilling, hypoxia and cyanamide exposure” up-regulate the expression of α-amylase genes in grapevine buds. J Plant Physiol. 2014;171(6):373–81. 10.1016/j.jplph.2013.11.009 24594388

[50] Gubler F, Raventos D, Keys M, Watts R, Jacobsen JV (1999). Target genes and regulatory domains of the *GAMYB* transcription activator in cereal aleurone. Plant J.

[51] Loreti E, Matsukura CA, Gubler F, Alpi A, Perata P (2000). Glucose repression of α-amylase in barley embryos is independent of *GAMYB* transcription. Plant Mol Biol.

[52] Lu C, Ho TD, Ho S, Yu S (2002). Three novel MYB proteins with one DNA binding repeat mediate sugar and hormone regulation of α-Amylase gene expression. Plant Cell.

[53] Xie Z, Zhang ZL, Zou X, Yang G, Komatsu S (2006). Interactions of two abscisic-acid induced *WRKY* genes in repressing gibberellin signaling in aleurone cells. Plant J.

[54] Schoonheim PJ, Pereira DDAC, Boer AHD (2009). Dual role for 14-3-3 proteins and ABF transcription factors in gibberellic acid and abscisic acid signalling in barley (*Hordeum vulgare*) aleurone cells. Plant Cell Environ.

[55] Ruan YL (2014). Sucrose metabolism: gateway to diverse carbon use and sugar signaling. Annu Rev Plant Biol.

[56] Xiong Y, Sheen J (2015). Novel links in the plant TOR kinase signaling network. Curr Opin Plant Biol.

[57] Yadav UP, Ivakov A, Feil R, Duan GY, Walther D, Giavalisco P, Piques M, Carillo P, Hubberten HM, Stitt M, lunn JE (2014). The sucrose-trehalose 6-phosphate (Tre6P) nexus: specificity and mechanisms of sucrose signalling by Tre6P. J Exp Bot.

[58] Liu Z, Shi Y, Xue Y, Wang X, Huang Z, Xue J, Zhang X (2021). Non-structural carbohydrates coordinate tree peony flowering both as energy substrates and as sugar signaling triggers, with the bracts playing an essential role. Plant Physiol Bioch.

[59] Carrillo N, Ceccarelli EA (2010). Open questions in ferredoxin-NADP^+^ reductase catalytic mechanism. FEBS J.

[60] Wang H, Liu Y, Yuan J, Zhang J, Han F (2020). The condensin subunits SMC2 and SMC4 interact for correct condensation and segregation of mitotic maize chromosomes. Plant J.

[61] Lyubimov AY, Costa A, Bleichert F, Botchan MR, Berger JM (2012). ATP-dependent conformational dynamics underlie the functional asymmetry of the replicative helicase from a minimalist eukaryote. Proc Natl Acad Sci USA.

[62] Thirugnanasambantham K, Prabu G, Mandal A (2020). Synergistic effect of cytokinin and gibberellins stimulates release of dormancy in tea (*Camellia sinensis* (L.) O. Kuntze) bud. Physiol Mol Biol Pla.

[63] Kojori ZK, Rezaei M, Sarkhosh A, Gharangik S (2018). The effect of bud-scale removal and gibberellin (GA_3_) on dormancy break of apricot (*P. Armeniaca* L.) vegetative buds. J Appl Horticult.

[64] Zhuang W, Gao Z, Wen L, Huo X, Cai B, Zhang Z (2015). Metabolic changes upon flower bud break in Japanese apricot are enhanced by exogenous GA_4_. Hortic Res-England.

[65] Grabherr MG, Haas BJ, Moran Y, Levin JZ, Thompson DA, Ido A, Xian A, Lin F, Raktima R, Qiandong Z (2011). Full-length transcriptome assembly from RNA-Seq data without a reference genome. Nat Biotechnol.

[66] Pertea G, Huang X, Liang F, Antonescu V, Sultana R, Karamycheva S, Lee Y, White J, Cheung F, Parvizi B (2003). TIGR Gene Indices clustering tools (TGICL): a software system for fast clustering of large EST datasets. Bioinformatics.

[67] Benjamini Y, Yekutieli D (2001). The control of the false discovery rate in multiple testing under dependency. Ann Stat.

[68] Anders S, Huber W (2010). Differential expression analysis for sequence count data. Genome Biol.

[69] Livak KJ, Schmittgen TD (2001). Analysis of relative gene expression data using real-time quantitative PCR and the 2^(-△△Ct)^ method. Methods.

[70] Chen ML, Fu XM, Liu JQ, Ye TT, Hou SY, Huang YQ, Yuan BF, Wu Y, Feng YQ (2012). Highly sensitive and quantitative profiling of acidic phytohormones using derivatization approach coupled with nano-LC-ESI-Q-TOF-MS analysis. J Chromatography B.

[71] Sun S, Wang H, Xie J, Su Y (2016). Simultaneous determination of rhamnose, xylitol, arabitol, fructose, glucose, inositol, sucrose, maltose in jujube (*Zizyphus jujube* Mill.) extract: comparison of HPLC-ELSD, LC-ESI-MS/MS and GC-MS. BMC Chemistry.

[72] Hansen J, Moler I (1975). Percolation of starch and soluble carbohydrates from plant tissue for quantitative determination with anthrone. Anal Biochem.

[73] Zhang T, Yuan Y, Zhan Y, Cao X, Liu C, Zhang Y, Gai S (2020). Metabolomics analysis reveals Embden Meyerhof Parnas pathway activation and flavonoids accumulation during dormancy transition in tree peony. BMC Plant Biol.

