# Supplementary material for: GA3 is superior to GA4 in promoting bud endodormancy release in tree peony (Paeonia suffruticosa) and their potential working mechanism
Source: BMC Plant Biol. 2021 Jul 5;21:323. doi: 10.1186/s12870-021-03106-2 (PMC8256580; doi:10.1186/s12870-021-03106-2)
Supplement: Supplementary file 1 — Additional file 1: The statistical data of unigenes annotated in public database. [file 12870_2021_3106_MOESM1_ESM.docx]

**Additional file 1 The statistical data of unigenes annotated in public database**

| **Anno_Database** | **Annotated Number** | **300<=length<1 000 bp** | **length>=1 000 bp** |
| --- | --- | --- | --- |
| NR | 28 420 (94.62 %) | 3 637 (12.11 %) | 24 751 (82.41 %) |
| eggNOG | 27 609 (91.92 %) | 3 502 (11.66 %) | 24 076 (80.16 %) |
| Swissprot | 25 071(83.47 %) | 2 934 (9.77 %) | 22 105 (73.60 %) |
| GO | 23 361(77.78 %) | 2 855 (9.51 %) | 20 474 (68.17 %) |
| KOG | 18 379 (61.19 %) | 2 257 (7.51 %) | 16 099 (53.60 %) |
| KEGG | 13 155 (43.80 %) | 1 773 (5.90 %) | 11 367 (37.85 %) |
| Pfam | 23(0.08 %) | 6 (0.02 %) | 16 (0.05 %) |
