# Supplementary figures and images for: GA3 is superior to GA4 in promoting bud endodormancy release in tree peony (Paeonia suffruticosa) and their potential working mechanism
Source: BMC Plant Biol. 2021 Jul 5;21:323. doi: 10.1186/s12870-021-03106-2 (PMC8256580; doi:10.1186/s12870-021-03106-2)

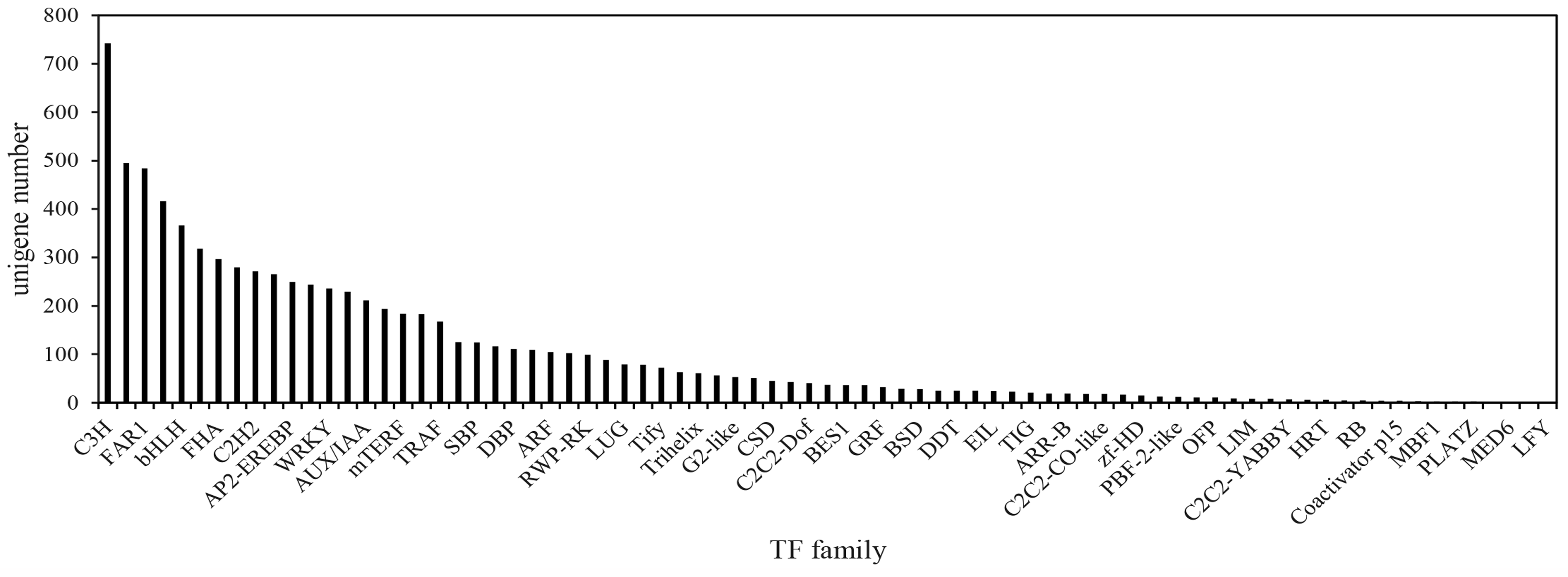

Supplement: Supplementary file 2 — Additional file 2: The number distribution of the unigenes encoding transcription factor (TF). [file 12870_2021_3106_MOESM2_ESM.tif]

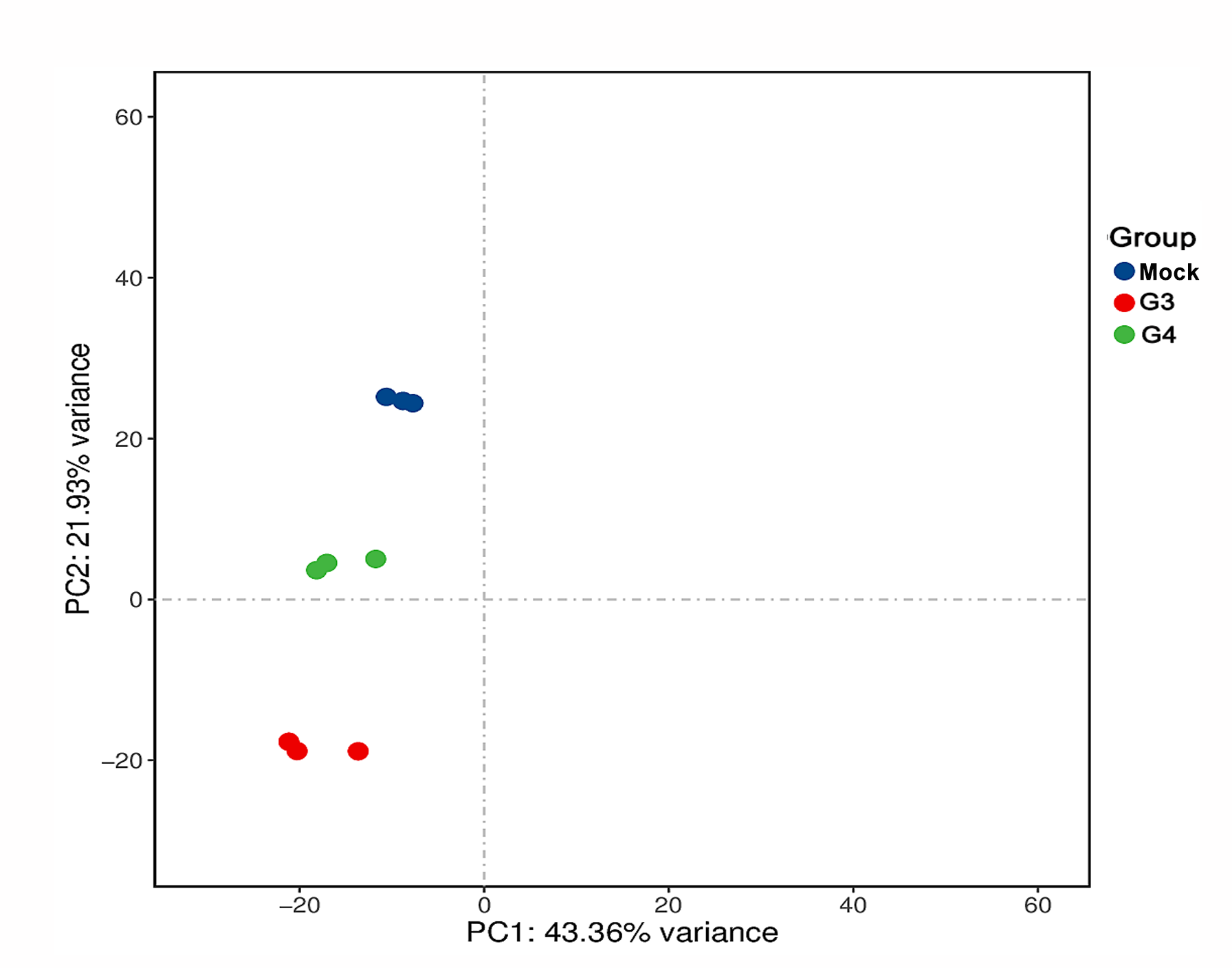

Supplement: Supplementary file 3 — Additional file 3: PCA analysis of the samples. [file 12870_2021_3106_MOESM3_ESM.tif]

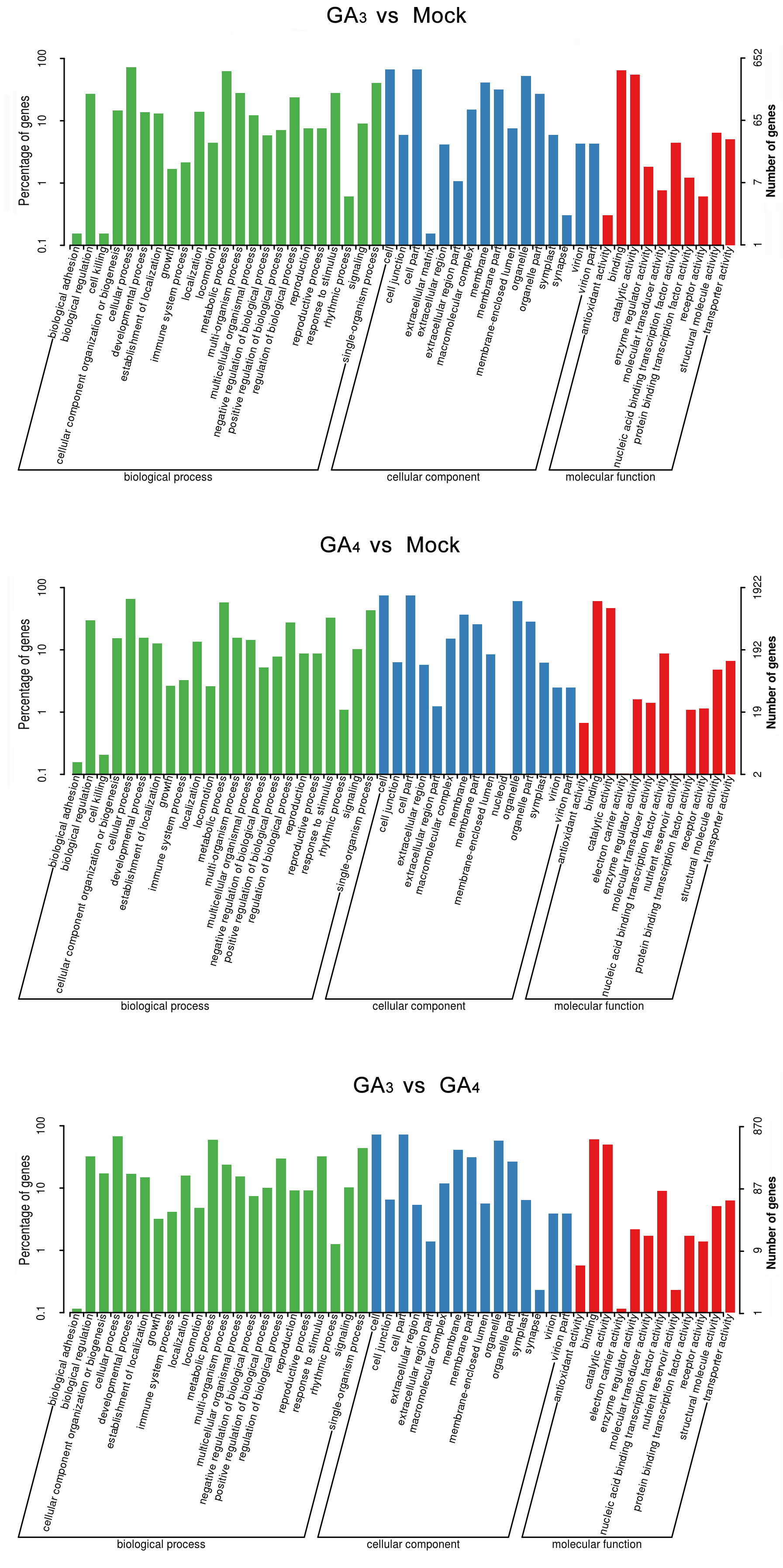

Supplement: Supplementary file 4 — Additional file 4: GO analysis of DEGs (p value<0.05 & |log2FC|>1) after GA3 and GA4 applications. [file 12870_2021_3106_MOESM4_ESM.tif]

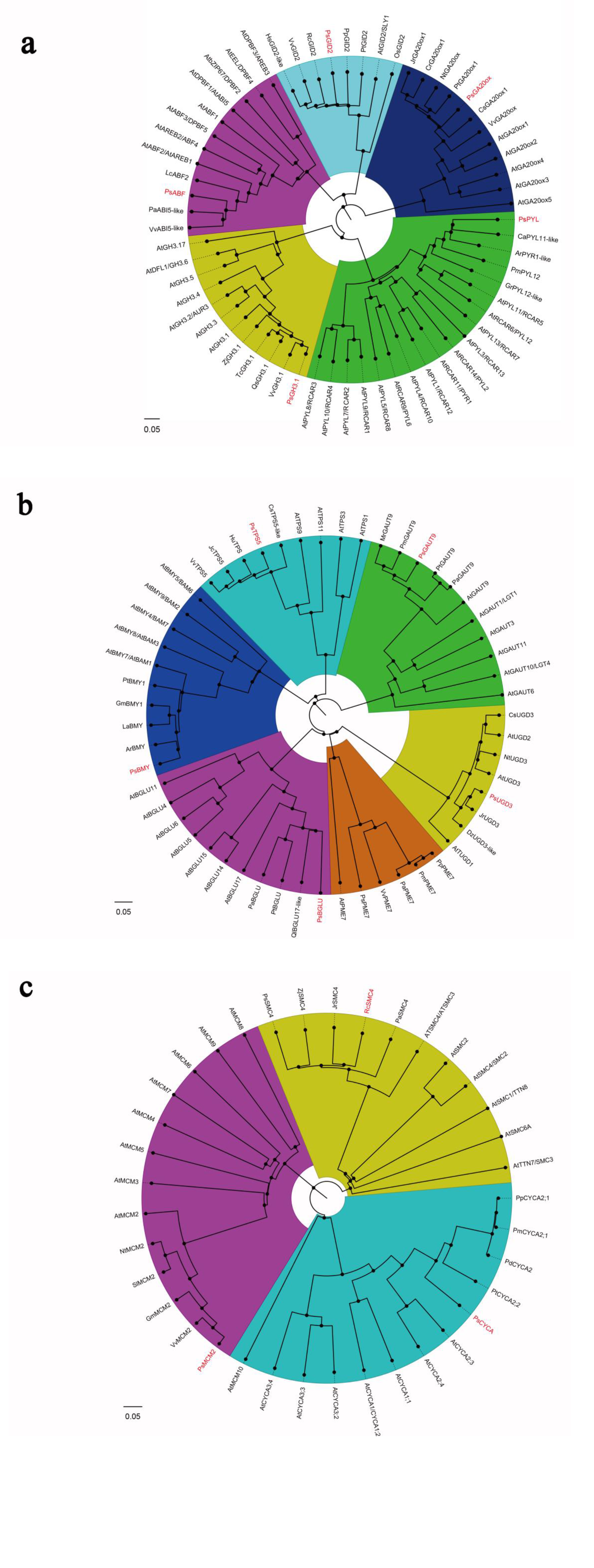

Supplement: Supplementary file 6 — Additional file 6: Based on Neighbor-Joining (NJ) model by MEGA 6.0, the phylogenetic trees of the related DEGs enriched in the KEGG pathways about plant hormone signal transduction, starch and sucrose metabolism, cell division and DNA replication. Genetic distance was calculated based on nucleotide difference (p-distance) with complete deletion of gaps. Scale bar = 0.05. The number at each node indicates the percentage of bootstrapping of 1000 replications. [file 12870_2021_3106_MOESM6_ESM.tif]

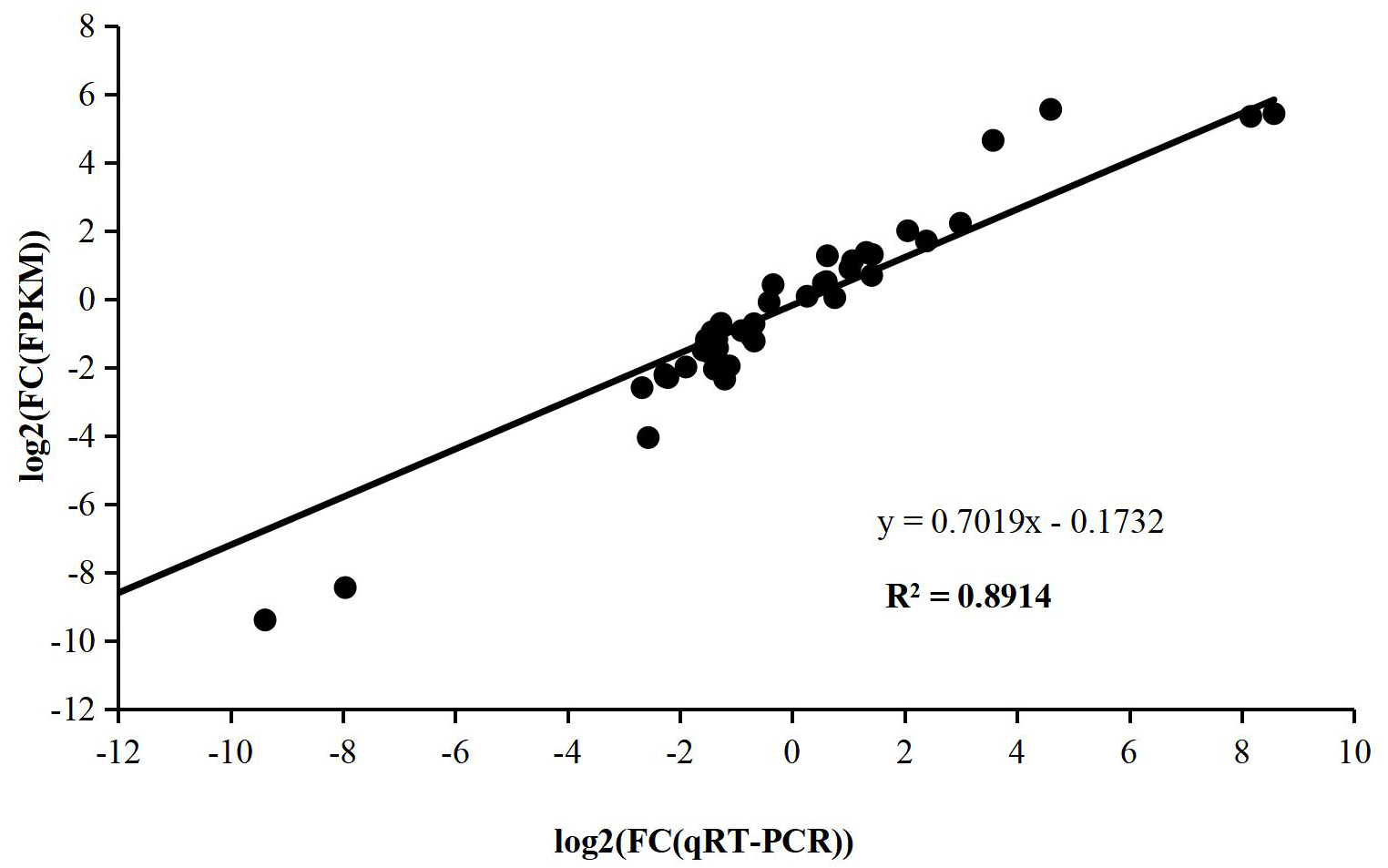

Supplement: Supplementary file 7 — Additional file 7: The correlation between the DEGs fold change of expression levels by qRT-PCR and the corresponding fold change with RNAseq. [file 12870_2021_3106_MOESM7_ESM.tif]
