# Supplementary material for: GA3 is superior to GA4 in promoting bud endodormancy release in tree peony (Paeonia suffruticosa) and their potential working mechanism
Source: BMC Plant Biol. 2021 Jul 5;21:323. doi: 10.1186/s12870-021-03106-2 (PMC8256580; doi:10.1186/s12870-021-03106-2)
Supplement: Supplementary file 8 — Additional file 8: Primer sequences used for qPCR analysis in this study. [file 12870_2021_3106_MOESM8_ESM.docx]

**Additional file 8 Primer sequences used for qPCR analysis in this study**

| Gene | Primer F (5'-3') | Primer R (5'-3') |
| --- | --- | --- |
| *PsGID2* | TTACTTTACGAGGTGCTCAAG | GAGGAAGAGGGCTTTGACAG |
| *PsGA20ox* | AGTGTTTGTAGACGATGAGTG | CCTGGGATTGTTATTAGTGTCC |
| *PsABF* | GGAAGACAGGAGGAACTGAGATT | TCCAAGACCACTGAAGGTGCT |
| *PsGH3.1* | CGAGCGTAAACAAGTCCTGAGA | ATCCCTTCCCAATCATCTTTCG |
| *PsPYL11* | GACAAGATGAATCACTTCCACAC | CTTGTGGTCACCGCCAATAAAG |
| *PsMYC2* | TCAAGAAACTCTCCAGCAACGC | ACTTCGTCATCCACCGTATCGT |
| *PsAMY* | TGATACTGGCTCAACACAGGC | GAGGATCGGCTGTGTAAGTCT |
| *PsBMY* | GAGAAGGATGCTGCTGAATG | ATTAAATAAGCCAAGCTATCT |
| *PsBGL* | TTTCTCTTGTGCGTGTTAGCC | CTGGATGCTTCTTGGTGAATG |
| *PsTPS5* | GCAGAGTGTTGTCTTGTTACGGC | TTCAGCCACAGCATCAATGTTC |
| *PsSMC4* | GGAGTAGACCTGGACAATAACCG | GCTTCTGCTTCATTCTTCACATC |
| *PsCYCA* | GCTAAAGTTGAAGAGCCCGCTA | TCACATAAGCATTCTCACAGCC |
| *PsFNR* | CCAACTGAGAAATCGTTCCGTA | CACCATAACTCTGTCCTTCCCAA |
| *PsMCM2* | CAACAAGTGTGATGCGGTCCTA | GCCTGTAACCTCAATCTCTTCC |
| *PsFT* | GGTTATGGTTGACCCTGATGC | CTCAGCGAAGTCTCTGGTGTTG |
| *PsTFL* | ACTGACATTCCTGGCACCAC | ACCAATGACTGGCTTTGGGA |
| *PsSVP* | GCACATGGTTTGCGAGGAAG | CCCGAATATGGCAGTCCCAA |
| *PsEBB1* | AATAGCCCGCGAAGTCCAAA | GGGATCTGATGAACCAGCCC |
| *PsEBB3* | GGTGAGATTACTCCGCCACC | CGACCCTGAATCTGAGACCG |
| *PsCYCD* | GAGGCCGTGGATTGGATTCT | AAAAGGGGCACTTGGGTCTC |
| *PsActin* | GAGAGATTCCGTTGCCCTGA | CTCAGGAGGAGCAACCACC |
